# Modulation of gene expression in drug resistant *Leishmania *is associated with gene amplification, gene deletion and chromosome aneuploidy

**DOI:** 10.1186/gb-2008-9-7-r115

**Published:** 2008-07-18

**Authors:** Jean-Michel Ubeda, Danielle Légaré, Frédéric Raymond, Amin Ahmed Ouameur, Sébastien Boisvert, Philippe Rigault, Jacques Corbeil, Michel J Tremblay, Martin Olivier, Barbara Papadopoulou, Marc Ouellette

**Affiliations:** 1 Université Laval, Division de Microbiologie, Centre de Recherche en Infectiologie, boulevard Laurier, Québec, G1V 4G2, Canada; 2 Université Laval, Centre de Recherche en Endocrinologie Moléculaire et Oncologique, boulevard Laurier, Québec, G1V 4G2, Canada; 3 McGill University, Department of Microbiology and Immunology, Lyman Duff Medical Building, University Street, Montreal, H3A 2B4, Canada

## Abstract

**Background:**

Drug resistance can be complex, and several mutations responsible for it can co-exist in a resistant cell. Transcriptional profiling is ideally suited for studying complex resistance genotypes and has the potential to lead to novel discoveries. We generated full genome 70-mer oligonucleotide microarrays for all protein coding genes of the human protozoan parasites *Leishmania major *and *Leishmania infantum*. These arrays were used to monitor gene expression in methotrexate resistant parasites.

**Results:**

*Leishmania *is a eukaryotic organism with minimal control at the level of transcription initiation and few genes were differentially expressed without concomitant changes in DNA copy number. One exception was found in *Leishmania major*, where the expression of whole chromosomes was down-regulated. The microarrays highlighted several mechanisms by which the copy number of genes involved in resistance was altered; these include gene deletion, formation of extrachromosomal circular or linear amplicons, and the presence of supernumerary chromosomes. In the case of gene deletion or gene amplification, the rearrangements have occurred at the sites of repeated (direct or inverted) sequences. These repeats appear highly conserved in both species to facilitate the amplification of key genes during environmental changes. When direct or inverted repeats are absent in the vicinity of a gene conferring a selective advantage, *Leishmania *will resort to supernumerary chromosomes to increase the levels of a gene product.

**Conclusion:**

Aneuploidy has been suggested as an important cause of drug resistance in several organisms and additional studies should reveal the potential importance of this phenomenon in drug resistance in *Leishmania*.

## Abstract 2

Gene expression and DNA copy number analyses using full genome oligonucleotide microarrays of *Leishmania* reveal molecular mechanisms of methotrexate resistance.

## Background

The protozoan parasite *Leishmania *is distributed worldwide and is responsible for a wide spectrum of diseases, including cutaneous, mucocutaneous and visceral leishmaniasis. No vaccines are presently available against *Leishmania *infections [1] and treatments rely primarily on chemotherapy. The chemotherapeutic arsenal is limited and resistance to the mainstay of pentavalent antimonials has reached epidemic proportions in parts of India [2]. Several studies dealing with drug resistance in *Leishmania *have highlighted the plasticity of the *Leishmania *genome [3,4]. The antifolate methotrexate (MTX) has been one of the first and most widely used drugs for understanding drug-induced plasticity and resistance mechanisms [5-8]. While *Leishmania *is sensitive to MTX, the drug is not used clinically to treat leishmaniasis. However, *Leishmania *is a folic acid auxotroph and studies of MTX resistance mechanisms have highlighted several novel aspects of folate metabolism in this parasite that could be exploited for drug interventions [9,10]. Indeed, the development of novel antifolate molecules for *Leishmania *and related parasites has been ongoing in several laboratories [11-13].

*Leishmania *resists MTX by a number of mechanisms. *Leishmania *has the capacity to transport folic acid, but this activity is often impaired in MTX resistant cells [8,14-17]. The main *Leishmania *folate transporter FT1 has been isolated [18,19] and is part of a large family of folate biopterin transporter (FBT) proteins with 14 members in *Leishmania *(AA Ouameur *et al*., unpublished data). Rearrangements of *FBT *genes are correlated with MTX resistance [19-21]. A frequent mechanism of drug resistance in *Leishmania *is gene amplification [3]. Small chromosomal regions of 20-70 kb that are part of one of the 36 *Leishmania *chromosomes are amplified as part of extrachromosomal elements [3]. These elements are usually formed by recombination between repeated homologous sequences [22-24]. Amplification of the gene coding for the target dihydrofolate reductase-thymidylate synthase (DHFR-TS) has been described in MTX resistant parasites [5,6,25-29]. Work on MTX resistance also led to the characterization of the pteridine reductase PTR1, whose main function is to reduce pterins. However, when overexpressed it can also reduce folic acid and lead to MTX resistance by by-passing DHFR-TS activity [30-33]. The *PTR1 *gene is frequently amplified as part of extrachromosomal circular or linear amplicons [6,16,22,34-38]. In addition to these three main mechanisms of resistance, perturbation in folate metabolism [39,40], in one carbon metabolism [41] or in DNA metabolism [42] have also been associated with MTX resistance. Several of these mutations can co-exist in the same cell, demonstrating that resistance can be a complex multi-gene phenomenon. Genome wide expression profiling scans represent a useful tool for understanding complex resistance mechanisms and may lead either to the discovery of novel resistance mechanisms and/or could provide clues about mechanisms of gene rearrangements.

Indeed, DNA microarrays have been useful for investigating the mode of action of drugs [43] and mechanisms of resistance (reviewed in [44-46]). DNA microarrays for *Leishmania *have evolved from random genomic DNA clones [47-50], cDNA clones [51,52], targeted PCR fragments [29], selected 70-mer oligonucleotides [53,54] to full genome microarrays [55,56]. Targeted microarrays have been used previously for the study of drug resistance in *Leishmania *[29,52,54,57]. We present here the generation of full genome DNA microarrays for both *L. major *and *L. infantum *and their use in the study of one *L. major *and one *L. infantum *MTX resistant mutant. These genome wide expression profiling experiments illustrate the complexity of resistance mechanisms present in the same cell. They allowed the definition of the precise mechanisms leading to the formation of extrachromosomal circular and linear amplicons, the definition of gene deletion events and revealed the involvement of aneuploidy in the complex genotype of MTX resistance.

## Results

### RNA expression profiling in methotrexate resistant *Leishmania *cells

**Figure 1:**
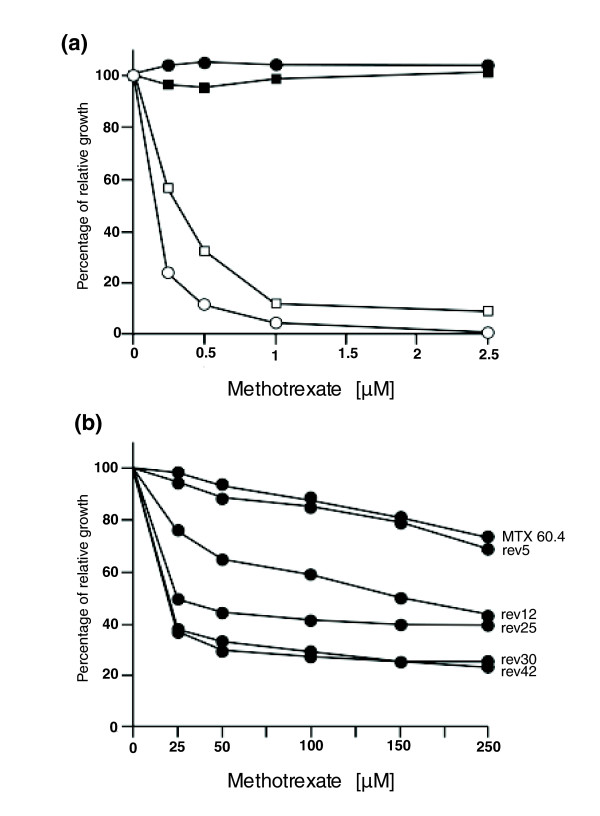
Methotrexate susceptibility in *Leishmania *cells. **(a) ***Leishmania *cells were grown in M199 medium and their growth was monitored at 72 hours by measuring their OD_600 nm _with varying concentrations of MTX. White circles, *L. major *wild-type cell; black circles, *L. major *MTX60.4; white squares, *L. infantum *wild-type cells; black squares, *L. infantum *MTX20.5. **(b) **The mutant *L. major *MTX60.4 was grown in the absence of drug for 5, 12, 25, 30 and 42 passages. The average of triplicate measurements is shown.

Completion of the *L. major *genome has allowed the generation of arrays containing 60-mer oligonucleotide probes designed by NimbleGen Systems [55,56] and in this work, we present the generation of a full genome DNA microarray composed of 70-mer oligonucleotide probes suitable for both *L. major *and *L. infantum *analysis (see Materials and methods for a full description of the arrays). These full genome arrays were used for deciphering how *Leishmania *resists the antifolate model drug MTX. Two MTX resistant mutants, *L. major *MTX60.4, which has previously been studied with small targeted arrays [29], and *L. infantum *MTX20.5, were studied using the full-genome microarrays. Mutants of both species are highly resistant to MTX (Figure 1a), and since they were selected in a stepwise fashion, it is likely that multiple resistance mechanisms may exist in these mutants and could thus be uncovered by these arrays. The resistant cells had a similar generation time as the wild-type parent cells.

**Figure 2:**
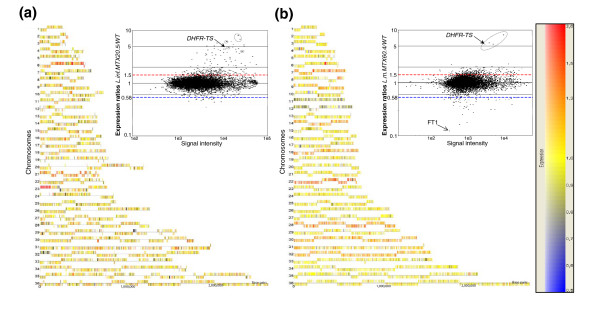
Modulation of gene expression in *Leishmania *cells resistant to methotrexate. DNA microarrays were analyzed as described in Materials and methods and the software GeneSpring version GX3.1 was used to represent fold modulation either on a chromosome by chromosome basis (1 to 36) or as a scatter plot (inserts) for both **(a) ***L. infantum *MTX20.5 and **(b) ***L. major *MTX60.4. Vertical bars refer to individual genes on each chromosome and their location above or below the strand represents the transcribed strand. Transcription in *Leishmania *leads to polycistronic RNAs. Red (increased expression) and blue (decreased expression) dashed lines in the scatter plots indicate 1.5-fold differences in gene expression, with the y-axis representing the expression ratios between the mutant and wild-type cells and the x-axis the signal intensity in the mutant. The color scale indicates the modulation of hybridization signals in the resistant mutants compared to wild-type cells. The spots corresponding to genes that are part of the *DHFR-TS *amplicons are circled in the scatter plots. The entire data set was deposited in GEO under the accession number series GSE9949.

**Figure 3:**
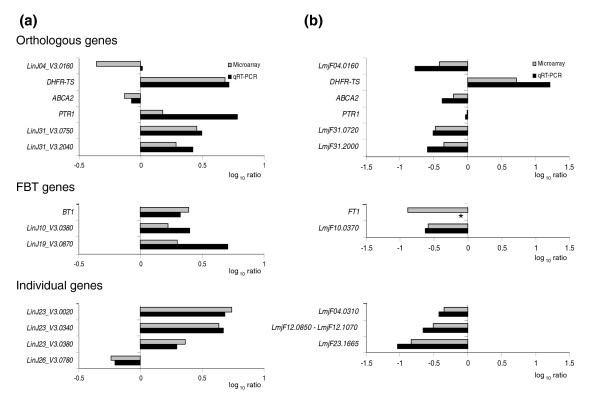
Validation of DNA microarray expression data by qRT-PCR. The mean log10 ratios of selected genes from microarray expression data (grey bars) are compared to qRT-PCR data (black bars) for **(a) ***L. infantum *MTX20.5 and **(b) ***L. major *MTX60.4. The microarray data are the average of four biological replicates (with two dye swaps), while the qRT-PCR data are the average of three biological replicates repeated two times each. The asterisk indicates that the related gene transcript was not detected by qRT-PCR. The upper panel shows the expression of orthologous genes where the expression changes in the two species; the middle panel shows the modulation in the expression of FBT genes; the lower panel shows the expression of individual genes specific for each mutant.

The DNA microarrays were first validated by hybridizing fluorescently labeled digested DNA of wild-type *L. major *and *L. infantum *cells. The arrays were found to yield uniform and reproducible results (not shown) and were deemed appropriate for RNA expression profiling experiments. Total RNAs were thus purified for both wild-type and mutant strains, used to synthesize fluorescent probes, and hybridized to the microarrays as described in Materials and methods. Scanning and normalization led to expression data that were first represented as scatter plots. As evident from these plots (inserts in Figure 2a,b), most genes in both species are equally expressed between the sensitive and resistant strains. Indeed, the bulk of expression (RNA level) ratios between sensitive and resistant strains were close to 1. Nonetheless, there were notable differences. First, the RNA levels of a total of 61 genes were found to be modulated (cut-off of 2, *p *< 0.05) in the *L. infantum *MTX20.5 mutant compared to the wild-type strain (Figure 2a; Table S1 in Additional data file 1) and the expression levels of 75 genes were changed significantly (cut-off of 2, *p *< 0.05) in the *L. major *MTX60.4 mutant compared to the wild-type strain (Figure 2b; Table S1 in Additional data file 1). Secondly, a majority of genes whose expression was modulated by more than two-fold had increased expression levels in *L. infantum *MTX20.5 but the majority of another set of genes had decreased expression levels in *L. major *MTX60.4 (inserts of Figure 2; Table S1 in Additional data file 1). If the expression modulation cut-off was changed from 2 to 1.5 (*p *< 0.05), we found 251 and 372 genes that were differentially expressed in *L. infantum *MTX20.5 and *L. major *MTX60.4, respectively (Figure 2). Surprisingly, few differentially expressed genes were found to be modulated similarly in both mutants (Figure 3; Table S1 in Additional data file 1). One notable exception is a region of chromosome 6 that corresponds to a six gene locus including the *DHFR-TS *gene. DHFR-TS is the main target for MTX and its gene was frequently found amplified in *L. major *MTX resistant mutants as part of extrachromosomal circles (reviewed in [3,4]).

The DNA microarray data were supported by selected quantitative real-time reverse transcription PCR (qRT-PCR) assays in both the *L. major *and *L. infantum *mutants (Figure 3). In only two cases we found a discrepancy between the two techniques. *LmjF04.0160 *and its orthologue *LinJ04_V3.0160 *were found down-regulated in both mutants using DNA microarrays, but this was confirmed only in the *L. major *mutant by qRT-PCR (Figure 3). The other discrepancy between microarray and qRT-PCR data was for *FT1*, but this is explained by a gene deletion event (see below). The only other gene that was modulated similarly in the two mutants was the ABC protein gene *ABCA2 *and this was confirmed by qRT-PCR (Figure 3). Other genes were modulated in both mutants but in different ways. While *LmjF31.0720 *was down-regulated in *L. major *MTX60.4, its orthologue *LinJ31_V3.0750 *in *L. infantum *MTX20.5 was overexpressed (Figure 3). Otherwise, genes differentially expressed were specific to individual mutants.

The differential gene expression of the MTX resistant mutants was also represented in a chromosome by chromosome fashion (Figure 2). This has permitted us to visualize regions that are differently expressed (red/orange, corresponding to overexpressed genes in the mutants). Two regions were clearly overexpressed in the *L. infantum *MTX20.5 mutant. One region was on chromosome 6 (*DHFR-TS *loci) and the second was in the left portion of chromosome 23 (Figure 2a). For the *L. major *MTX60.4 mutant, we also saw an increase in expression of selected genes present on chromosome 6 (*DHFR-TS *loci), but we also observed a number of whole chromosomes (for example, chromosome 22; colored predominantly red in Figure 2b).

### Extrachromosomal circular amplification of *DHFR-TS*

**Figure 4:**
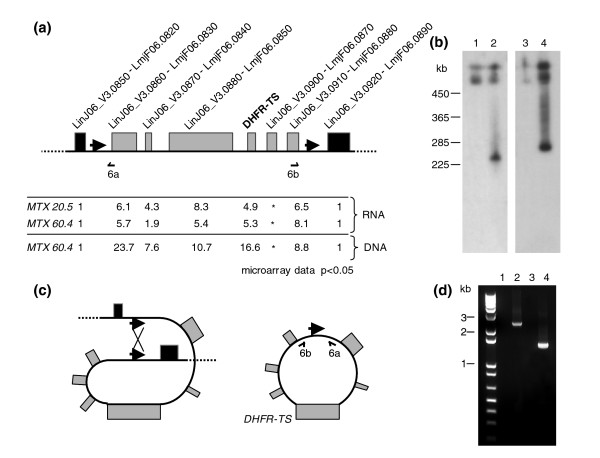
Extrachromosomal circular amplification of a genomic region of *Leishmania *chromosome 6 that includes the *DHFR-TS *locus. **(a) **Genomic organization of the *DHFR-TS *locus in both *L. infantum *MTX20.5 and *L. major *MTX60.4. Relative gene expression data (RNA) were determined using DNA microarrays and relative hybridization data were obtained by comparative genomic hybridization (DNA). Asterisks indicate that the microarray data of these genes were not found to be reliable. Direct repeats are shown with thick arrows and the approximate position of primers 6a and 6b are indicated with half arrows. **(b) **Chromosome size blot of *Leishmania *cells hybridized to a *DHFR-TS *probe. Sizes were determined using a yeast molecular weight marker (Biorad. Hercules, CA, USA). **(c) **Model for the formation of the extrachromosomal *DHFR-TS *circular DNA generated through homologous recombination between direct repeats (Figure S1 in Additional data file 2). **(d) **PCR with primers 6a and 6b to support the model shown in (c). Lane 1, *L. infantum *wild-type cells; lane 2, *L. infantum *MTX 20.5; lane 3, *L. major *wild-type cells; lane 4, *L. major *MTX60.4.

*DHFR-TS *is present on chromosome 6 and by close examination of the expression data derived from the arrays we were able to precisely define the genes with increased expression in both the *L. major *and *L. infantum *mutants. In *L. infantum*, the genomic region overexpressed is delimited by genes *LinJ06_V3.0860 *and *LinJ06_V3.0910 *(Figure 4a). Most interestingly, the same region is overexpressed in *L. major *MTX60.4 (Figure 4a). As *Leishmania *is devoid of control for the initiation of transcription (no pol II promoter has yet been isolated in this parasite [58]), it is possible that the amplification of a small genomic region containing the *DHFR-TS *gene is responsible for the increased gene expression as determined by DNA microarrays. This was tested by hybridization of a blotted pulsed-field gel electrophoresis (PFGE) gel with a *DHFR *probe. Wild-type cells gave rise to two hybridizing bands, suggesting that the two homologous chromosomes 6 have different sizes (Figure 4b, lanes 1 and 3), a well established phenomenon in *Leishmania *[59]. The two mutants had an extra band hybridizing to the *DHFR *probe, which with its hybridizing smear is characteristic of extrachromosomal circles (Figure 4b, lanes 2 and 4). The genesis of circular DNA in *Leishmania *has been studied and is often due to homologous recombination between direct repeats bordering the regions amplified [22-24]. Close examination of the sequences flanking the regions amplified indeed pointed to the presence of repeated sequences (Figure 4a). The repeated sequences were highly similar between *L. major *(575 bp) and *L. infantum *(837 bp) (Figure S1 in Additional data file 2). To provide evidence that the *DHFR-TS *containing circles were generated through homologous recombination between these direct repeated sequences, we used two primers (6a and 6b in Figure 4a,c) that should give rise to a PCR amplification product only when an extrachromosomal circle is formed (Figure 4c). Indeed, when using this primer pair, PCR fragments of the expected size were observed in *L. infantum *MTX20.5 and *L major *MTX60.4 (Figure 4d, lanes 2 and 4) while no amplification was observed in the wild-type cells (Figure 4d, lanes 1 and 3). The difference in size of the PCR fragments between *L. major *and *L. infantum *is due to the difference in size of the repeats in the two species (Figure S1 in Additional data file 2). Sequencing of the PCR generated amplicon derived from *L. major *MTX60.4 [GenBank:EU346088] confirmed the scenario of homologous recombination between the repeated sequences (Figure S1d in Additional data file 2).

### Linear amplification of *PTR1*

**Figure 5:**
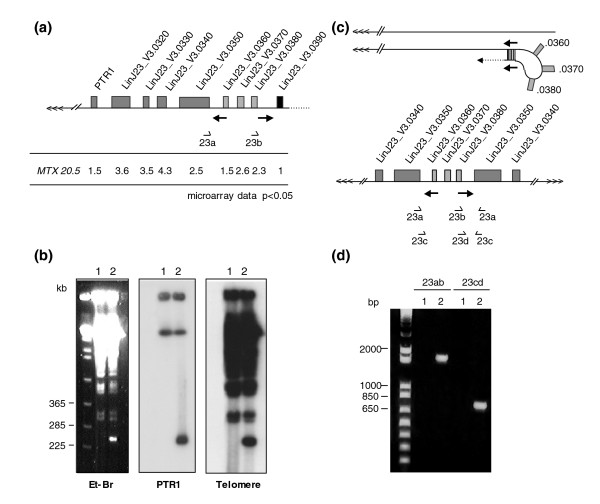
Linear amplification of *PTR1 *as a large inverted duplication. **(a) **Genomic organization of the *PTR1 *locus in *L. infantum *and relative gene expression data as determined by DNA microarrays in *L. infantum *MTX20.5. Note that all genes from the telomere up to *LinJ23_V3.0380 *showed increased levels of expression in the MTX20.5 mutant compared to wild-type cells. **(b) **Chromosome size PFGE of *Leishmania *cells. Ethidium bromide (Et-Br) stained gel, or blotted gels hybridized to a *PTR1 *probe or to a probe containing the telomeric repeats are shown. Sizes were determined using a yeast molecular weight marker (Biorad). **(c) **Model for the formation of the extrachromosomal *PTR1 *linear amplicon generated through annealing of homologous inverted repeats (Figure S2 in Additional data file 2). This annealing could be facilitated by a block in replication. **(d) **PCR with primer pairs 23a and 23b or 23c and 23d to support the model shown in (c). Lane 1, *L. infantum *wild-type cells; lane 2, *L. infantum *MTX20.5.

In mutant *L. infantum *MTX20.5 we observed a region of chromosome 23 that was overexpressed (increased RNA levels; Figure 2a). This region contains the gene for pteridine reductase 1 (*PTR1*), a well established MTX resistance gene whose product can reduce folic acid, hence by-passing the need for DHFR-TS [30,31]. Similarly to the *DHFR-TS *loci, the microarray expression data have allowed the precise determination of the region that was overexpressed, which started at the telomeric end and extended 120 kb up to gene *LinJ23_V3.0380 *(Figure 5a). The putative presence of telomeric sequences would suggest a linear amplification instead of a circular amplification. Hybridization of a chromosome PFGE blot has shown that *PTR1 *hybridized to the approximately 800 kb chromosome in both wild-type and resistant cells but also to a smaller linear amplicon of approximately 230 kb in *L. infantum *MTX20.5 (Figure 5b). This amplicon also hybridized to a telomere probe (Figure 5b). The size of the amplicon suggests that the amplified region was duplicated. The *LinJ23_V3.0390 *gene is clearly not overexpressed and thus not part of the amplicon (Figure 5a). Three genes, *LinJ23_V3.0360*, *LinJ23_V3-0370 *and *Lin23_V3.0380*, were less overexpressed than the other genes that are part of the amplicon (Figure 5a). Examination of the sequences where expression changed enabled the detection of inverted homologous repeats of 578 bp (Figure S2 in Additional data file 2) between *LinJ23_V3.0350 *and *Lin23_V3.0360*, and between *LinJ23_V3.0380 *and *Lin23_V3.0390 *(Figure 5a). Interestingly, similar repeats of 574 bp with 91% identity were found at the same position in the *L. major *genome [60]. The presence of these inverted repeats and the microarray expression data would suggest the formation of a linear amplicon with large inverted duplications that was formed by annealing of the identical 578 bp inverted repeats (Figure 5c). To obtain support for this scenario, we used PCR primer pairs (23a and 23b, or 23c and 23d) that would lead to a PCR product only if the rearrangement had occurred at the level of the inverted repeats (as, for example, during a block in DNA replication). Indeed, we obtained a product of the expected size with these pairs of primers in *L. infantum *MTX20.5 but no product was obtained from DNA derived from wild-type cells (Figure 5d). The nucleotide sequence of the PCR amplicon obtained with primer pair 23a/23b [GenBank:EU346089] is entirely consistent with the model shown in Figure 5c (Figure S2 in Additional data file 2).

### Decrease in gene expression due to deletion of folate transporter genes

**Figure 6:**
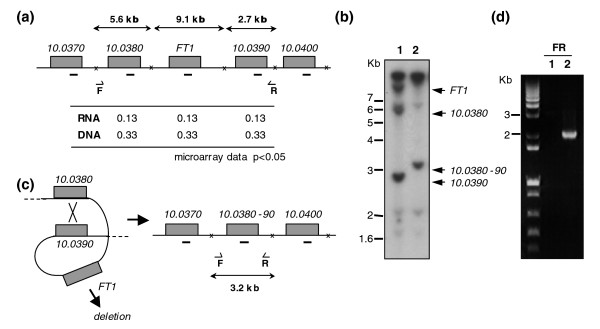
Mechanism of deletion of the main folate transporter gene *FT1 *in *L. major *selected for MTX resistance. **(a) **A portion of the *L. major *chromosome 10 showing some of the FT genes. Approximate location of *Pvu*I sites (crosses) and their size are shown. Primers F and R are indicated by half arrows. The relative hybridization data obtained from RNA expression profiling (RNA) and comparative genomic hybridization (DNA) are shown. Due to conservation between the FT genes, the 70-mer probes for *LmjF10.0380*, *FT1 *and *LmjF10.0390 *are not discriminatory. **(b) **Southern blot of *Leishmania *total DNA digested with *Pvu*I and hybridized to a probe recognizing conserved sequences of most *FBT *genes (indicated by bars underneath the genes in (a,c)). The genes corresponding to some hybridizing bands are indicated. **(c) **Model for the deletion of *FT1 *mediated by the homologous recombination of the conserved sequences between the folate transporter genes *LmjF10.0380 *and *LmjF10.0390 *(Figure S3 in Additional data file 2). **(d) **PCR with primers F and R to support the model shown in (c). Lane 1, *L. major *wild-type cells; lane 2, *L. major *MTX60.4.

*Leishmania *spp. have a large gene family of conserved folate transporters with 14 FBT members (AA Ouameur *et al*., unpublished data). Part of this family located on chromosome 10 is shown in Figure 6a. Microarray expression data indicated that *FT1*, coding for the main *Leishmania *folate transporter [18,19], is down-regulated in *L. major *MTX60.4 but not in *L. infantum *MTX20.5 (Figure 3). The level of conservation of the various FBTs precluded that the 70-mer oligonucleotides spotted on the arrays would discriminate several of these closely related genes. The use of qRT-PCR to confirm the microarray data indicated that *FT1 *may be absent (Figure 3). This was suggestive of a gene deletion event and indeed a Southern blot of *L. major *MTX60.4 DNA hybridized with a probe recognizing the majority of *FBT *genes confirmed this extensive gene rearrangement (Figure 6b) and bands corresponding to *LmjF10.0380*, *LmjF10.0385 *(*FT1*) and *LmjF10.0390 *were either lacking or rearranged. Using PCR primers (labeled F and R in Figure 6a,c), we were able to demonstrate that *FT1 *(*LmjF10.0385*) was deleted following an event of homologous recombination between conserved sequences between *LmjF10.0380 *and *LmjF10.0390 *(Figure 6c). Indeed, primers F and R gave rise to a PCR fragment of 2.2 kb in *L. major *MTX60.4 (Figure 6d, lane 2) while under the conditions tested no fragments were found with *L. major *wild-type cells. Sequencing of the amplicon [GenBank:EU346090] validated the scenario of homologous recombination between two *FBT *genes leading to the diploid deletion of *FT1 *(Figure 6c; Figure S3 in Additional data file 2).

### Selection for MTX resistance and chromosome aneuploidy

**Figure 7:**
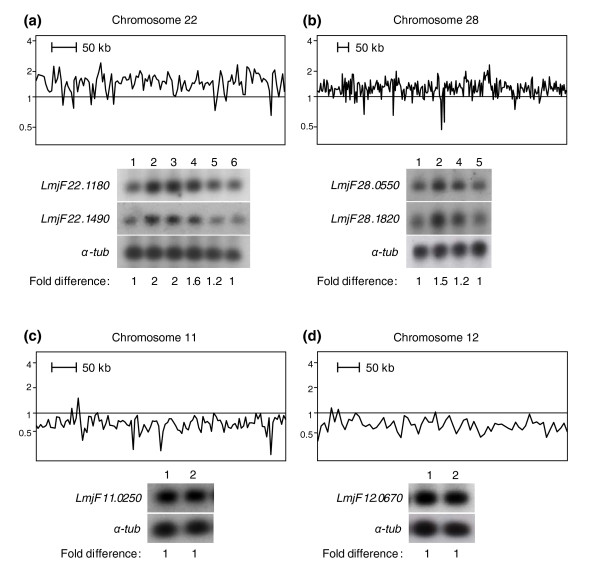
Chromosome aneuploidy in *L. major *selected for MTX resistance. The relative expression ratio of each individual gene of chromosomes **(a) **22, **(b) **28, **(c) **11 and **(d) **12 of *L. major *MTX60.4 was contrasted with the expression levels of the same genes *in L. major *wild-type cells, which were arbitrarily set at 1. Quantitative Southern blots were performed; two distant probes per chromosome were hybridized to *Hpa*II digested DNA from *L. major *wild-type (lane 1), and *L. major *MTX60.4 (lane 2) (only one hybridization is shown for chromosomes 11 and 12). The hybridization signals of an α-*tubulin *(α-*tub*) probe, whose related gene is unchanged in the resistant strain, were used to standardize all the hybridization signals. *Hpa*II digested total DNA from revertant *L. major *MTX60.4 parasites after 5, 12, 25, and 30 passages without MTX (lanes 3, 4, 5, and 6, respectively) were added, showing the progressive loss of aneuploid chromosomes in revertants.

Analysis of gene expression on a chromosome by chromosome basis (Figure 2) suggested that the expression of whole chromosomes is modulated in *L. major *MTX60.4. Indeed, the majority of genes present on chromosomes 11 and 12 appeared down-regulated while the expression of genes located on chromosomes 7, 22, 28 and 32 seemed up-regulated (Figure 2). Chromosome 6 of *L. infantum *MTX20.5 also appears to be in more than two copies. This chromosome-wide uniform modulation of expression was represented more thoroughly for selected chromosomes by plotting the fold modulation in gene expression along the chromosome (Figure 7). The normalized microarray data indicated that genes of chromosomes 22 and 28 were overexpressed 1.7- and 1.5-fold, respectively, in the resistant strain *L. major *MTX60.4 compared to the wild-type strain. The expression of genes on chromosomes 11 and 12 seemed, in general, to be 50% underexpressed in the mutant strain compared to wild-type cells (Figure 7).

A number of hypotheses can explain this whole chromosome-specific gene regulation and we tested whether the copy number of specific chromosomes changed upon MTX selection in *L. major *MTX60.4. Quantitative Southern blot analyses with two distinct probes derived from chromosome 22 revealed that if the wild-type cells contain two homologous copies of chromosome 22 (*Leishmania *is a diploid organism), *L. major *MTX60.4 had four copies (Figure 7a, lanes 1 and 2). Similarly, *L. major *MTX60.4 had three copies of chromosome 28 compared to wild-type cells (Figure 7b, lanes 1 and 2). The probes used are physically far apart, indicating a change in ploidy of the whole chromosome. However, this change in chromosome copy number was not observed for chromosomes 11 and 12 (Figure 7c,d). Aneuploidy of specific chromosomes and drug resistance has been described in cancer cells (reviewed in [61]) and fungi [62,63]. To test this possibility, we generated a revertant line of *L. major *MTX60.4 by successive passages in the absence of MTX; under these conditions, resistance to the drug decreased (Figure 1b). Revertant cells were not as sensitive as wild-type cells to MTX but this is expected as a deletion of *FT1 *(Figure 6) will lead to resistant parasites [19]. The aneuploidy of chromosomes 22 and 28 regressed to diploidy (similar to wild-type diploidy) after 30 passages, thus circumstantially linking resistance levels (Figure 1b) and copy number of these chromosomes (Figure 7a,b, lanes 2-6). With the cells now diploid, additional passages (for example, passage 42) did not decrease resistance further.

### Comparative genomic hybridization

**Figure 8:**
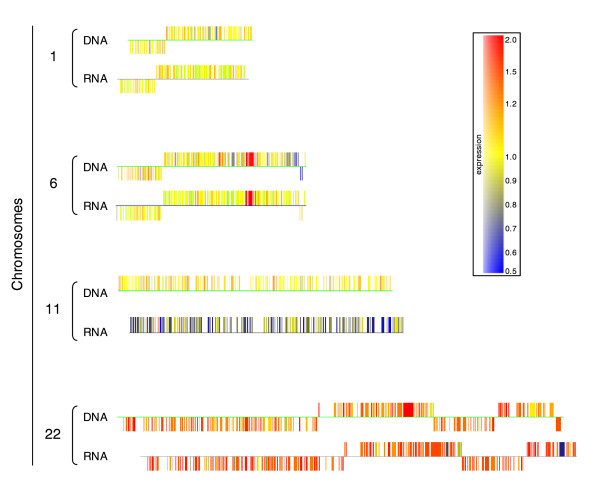
Comparison of relative hybridization data between RNA expression profiling and comparative genomic hybridization. RNA or genomic DNA derived probes were prepared from *L. major *MTX60.4 and the sensitive parent strain and hybridized to DNA microarrays. A subset of whole chromosome comparisons showing the correlation between RNA and DNA hybridization data are depicted. Examples shown are: chromosome 1 used as a no change control; chromosome 6 and the overexpression/amplification of the *DHFR-TS *locus (for quantification see Figure 4); and chromosome 22, where DNA and RNA are increased. For chromosome 11, RNA is decreased while DNA appears the same but the latter was also confirmed by Southern blots (Figure 7).

Since several of the changes in RNA levels were correlated with gene amplification or gene deletion, we undertook a comparative genomic hybridization (CGH) study using the full genome array. The DNA of mutant *L. major *MTX60.4 was labeled and changes in copy number in comparison to sensitive wild-type cells were measured using CGH. The CGH data are represented in a chromosome by chromosome fashion in Figure S4 in Additional data file 3. A qualitative correlation was observed between CGH and RNA-based hybridization (Figure 8). Indeed, amplification of the *DHFR-TS *locus, derived from chromosome 6, was easily detected by both techniques and quantification of the DNA amplification was compared to RNA levels (Figure 4). The deletion of *FT1 *was also detected by CGH and the latter technique was found to be quantitative. Indeed, the 70-mers recognizing *FT1 *recognized three conserved FT genes. In the MTX60.4 mutant two of these genes are deleted, hence explaining the ratio of 0.33 obtained by CGH (Figure 6). Polyploidy was also easily detected by CGH (Figure 8). Indeed, a similar qualitative pattern of hybridization intensities was obtained for both RNA expression profiling and CGH (Figure 8). Interestingly, while RNA expression profiling showed that chromosome 11 was down-regulated, quantitative Southern blots indicated that the copy number of the chromosome remained unchanged (Figure 7). This was also confirmed by CGH (Figure 8). There are some differences, however, between RNA expression profiling and CGH. For example, the latter technique showed that chromosome 2 is polyploid (Figure S4 in Additional data file 3) but this is likely due to the dynamic process of cell culture and parasite evolution, as DNA and RNA were prepared 1.5 years apart, rather than a difference in the techniques.

## Discussion

The use of DNA microarrays is now useful to understand both the mode of action of drugs and the mechanisms of drug resistance (reviewed in [44-46]). Since *Leishmania *has no control at the level of transcription initiation [58], it is unlikely that drug response profiling using microarrays will be helpful to understand the mode of action of drugs in *Leishmania*. Results using MTX as a lead drug and qRT-PCR to monitor key genes, such as *DHFR-TS*, *PTR1*, and *FT1*, appeared to confirm this lack of RNA modulation of target genes upon drug exposure (unpublished observations). This is unfortunate, as the mode of action of most anti-*Leishmania *drugs is unknown. Nonetheless, microarrays are likely to be useful for studying resistance in *Leishmania *since it is often mediated by gene amplification [3,4] and we show here that DNA arrays hybridized to cDNAs were most valuable for detecting gene amplification events (Figures 2, 4, and 5). Since resistance is mostly correlated with gene amplification, we also used CGH and found a good qualitative correlation between RNA expression profiling and CGH (Figure 8). The technique of CGH was found to be technically simpler, but since there are clear examples of modulation in RNA level (for example, increased RNA stability) without changes in copy number of DNA in drug resistant *Leishmania *[64-66] (Figure 3, and Figure 7 for chromosomes 11 and 12), hybridization with cDNAs is likely to be more comprehensive. Nonetheless, modulation in RNA levels without changes in copy number of a gene is an infrequent event in drug resistant *Leishmania*. The use of both *L. infantum *and *L. major *MTX resistant mutants validated the design of our multi-species array but has also illustrated that the cellular resistance genotype can be complex and differ considerably between different mutants selected for resistance to the same drug. The modulation in expression of a few genes was common to both mutants, and only *ABCA2 *and *DHFR-TS *could be confirmed by qRT-PCR (Figure 3). Down-regulation of the ABC protein gene *ABCA2 *has never been described in MTX resistant *Leishmania *cells and additional investigations would be required to test whether it has any role in MTX resistance.

*DHFR-TS *was the first amplified gene studied in a protozoan parasite [5] but its exact mechanism of amplification has never been reported. In addition to detecting gene amplification events, microarray data, whether derived from RNA expression profiling or CGH, were also useful in mapping the exact regions that were amplified. We show that *DHFR-TS *is amplified in *L. major *MTX60.4 as an extrachromosomal circle through homologous recombination between non-coding repeated sequences (Figure 4). This is consistent with other loci that were also found to be amplified by homologous recombination between relatively long repeated sequences [22-24]. Blast searches have shown that these exact repeated sequences are found only on chromosome 6. Remarkably, the same similar repeated sequences (albeit with different sizes) have also been conserved in *L. infantum *(Figure S1 in Additional data file 2). The same observation was made for the inverted repeats close to *PTR1 *that were conserved between *L. major *and *L. infantum*. *L. major *and *L. infantum *are thought to have diverged 0.5 million years ago [67] and it thus seems that there is considerable selective pressure to keep these repeated sequences intact. Since folates and pterins are important for *Leishmania *growth, it is possible that the presence of these repeats may allow a strategy to rapidly increase DHFR-TS or PTR1 levels in conditions of limited substrates. With its lack of transcription initiation control, *Leishmania *may utilize this alternative strategy of flanking key metabolic genes by repeated sequences to amplify these genes when required. Consistent with this proposal, DNA amplification has been observed in *Leishmania *cells subjected to nutrient shocks [68].

PTR1 is a well established MTX resistance gene product [30,31] and the amplification of its gene was first reported as part of extrachromosomal circles [6,34-36]. Linear amplification of *PTR1 *with inverted duplications was described later [16,24,37] and linear amplicons could be precursors of circular amplicons [38]. Linear amplicons derived from other loci than the *PTR1 *region with inverted duplications have also been described in *Leishmania *[69-73]. The microarray hybridization data have enabled the elaboration of a plausible model for the generation of a linear amplicon that contained large inverted duplications formed at the site of inverted repeats (Figure 5). This is consistent with other models of gene amplification in *Leishmania *[16,37] where inverted repeats seem to be a major pathway to generate amplified large DNA palindromes (inverted duplications), as described in *Tetrahymena *[74], yeast [75] and mammalian cancer cells [76,77]. One of the large inverted duplications extends from the inverted repeats, where rearrangement has occurred, to the telomeric sequences (Figure 5). These data exclude the necessity of chromosomal breaks/rearrangements at two independent positions, but it remains to be determined whether a double-stranded break, a single-stranded break or blocks in replication are facilitating inverted repeat annealing.

Gene deletions were thought to be associated with MTX resistance in *Leishmania *[19,20] but had not yet been characterized at the molecular level. The microarray data, either derived from RNA expression profiling or CGH, has led to the observation that a diploid non-conservative deletion occurred by homologous recombination between two members of the large *FBT *gene family (Figure 6). The mechanism of gene deletion thus resembles the mechanism of amplification. Usually, amplification in *Leishmania *is conservative, and only a few instances of non-conservative amplification (loss of one allele) have been described in it [3,22,23]. In the *L. major *MTX60.4 mutant, we observed a diploid deletion of the *FT1 *gene (Figure 6). It is not known whether the second allele is deleted by homologous recombination or by a gene conversion event such as a loss of heterozygosity, but there is a strong selection pressure to delete *FT1*, the main folate (and MTX) transporter in *Leishmania*. Without FT1, cells can become resistant to MTX but folates or related molecules will still need to be transported. It will be of interest to determine whether the fusion FBT protein produced by the recombination event (Figure 6) is active or not.

The microarray approach has shown that modulation of gene expression could (rarely) be due to differential RNA expression without changes in copy number (Figure 3) [29]; it could be more frequently due to gene amplification (Figures 4 and 5) and, as determined now, to gene deletion (Figure 6). Two novel strategies were highlighted through the use of microarrays. In the *L. major *MTX60.4 mutant, the entire set of genes of chromosomes 11 and 12 is down-regulated while all the genes present on chromosomes 22 and 28 and possibly a few other chromosomes are overexpressed. The mechanism underlying an upregulation in gene expression results from a change in chromosome ploidy (Figure 7). Changes in ploidy have been observed when attempting to inactivate essential genes in *Leishmania *[78], but not in resistant parasites. We recently observed a similar phenomenon with other resistant *Leishmania *cells (P Leprohon *et al*., unpublished data), suggesting that chromosome aneuploidy is part of the *Leishmania *arsenal for responding to drug pressure. There was a good correlation between resistance levels and the copy number of these supernumerary chromosomes (Figures 1 and 7), linking this genetic event to the resistance phenotype. Obviously, additional studies will be required to determine which gene(s) is (are) responsible for resistance. A putative mechanism for increasing the levels of a gene product in *Leishmania *would thus be to generate supernumerary chromosomes. This may occur when direct or inverted repeats are absent in the vicinity of a gene conferring a selective advantage. While this is plausible, especially for an organism lacking control at the level of transcription initiation, this drug induced aneuploidy has been well documented in cells with transcriptional control, such as cancer cells (reviewed in [61]) or fungi [62,63]. The mechanism of down-regulation of whole chromosome expression does not seem to involve a change in chromosome number (Figures 7 and 8) and may involve epigenetic factors that will need to be investigated.

## Conclusion

The microarray approach was useful in highlighting several mechanisms used by resistant cells to modulate the copy number of genes by: gene deletion or extrachromosomal circular or linear amplicons; through supernumerary chromosomes; and by decreasing the expression of whole chromosomes by a mechanism that remains to be identified. In the case of the first two events, the rearrangements have occurred at the site of repeated (direct or inverted) sequences. It is possible that these repeats are not randomly distributed to allow the amplification of specific chromosomal regions. Using DNA microarrays it was shown that inverted duplications are frequent in cancer cells; these are not randomly distributed, and a subset are associated with gene amplification [79]. The availability of DNA microarrays for *Leishmania *has highlighted the role of repeated sequences and of chromosome ploidy in responding to environmental changes. Aneuploidy has been suggested as an important cause of cancer specific drug resistance [61] and further work should reveal the potential importance of this phenomenon in drug resistance in *Leishmania*.

## Materials and methods

### Cell culture

The wild-type strain *L. major *LV39 and the mutants *L. major *MTX60.4 have been described previously [65]. The *L. infantum *strain (MHOM/MA/67/ITMAP-263) was selected *in vitro *in a stepwise fashion starting with its EC_50 _(0.5 μM) with doubling concentrations of MTX when cells were adapted to yield *L. infantum *MTX20.5 growing at 20 μM of MTX. All cells were grown in M199 medium supplemented with 10% heat-inactivated fetal bovine serum and 5 μg/ml hemin at 25°C.

### DNA manipulation

Chromosomes in agarose blocks were prepared and separated by PFGE as described previously [38]. For Southern blot and PCR, genomic DNA was isolated using the DNAzol technique (Invitrogen, Carlsbad, CA, USA) as recommended by the manufacturer. Southern blots, hybridization, and washing conditions were done following standard protocols [80]. For chromosome copy number investigation, Southern spots were quantified using ImageQuant 5.2 (GE Healthcare, Upsala, Sweden) and the reference gene *α-tubulin *was used for normalization.

### *L. infantum *and *L. major *DNA oligonucleotides full genome microarray design

The recent completion of the sequence of the *L. major *[81] and *L. infantum *[82] genomes, allowed the generation of multispecies high-density oligonucleotide microarrays. Our analysis of open reading frame sequence conservation between *L. major *and *L. infantum *revealed that these two species share 91-96% nucleotide identity, suggesting that interspecies microarray probes can be designed. Therefore, 70-mer oligonucleotides were designed for each open reading frame of *L. infantum *and *L. major *using automated bioinformatic procedures. The genomes of both species were first compared using BLAST and homologous genes were grouped together. Probes were designed with consistent thermodynamic properties. Probes were initially designed for *L. infantum *with the added requirement that the region targeted by the probes had perfect homology between both species. For common probes, up to 2 mismatches (out of 70 nucleotides) were tolerated. In the case that more than two mismatches were present in a given gene between *L. infantum *and *L. major*, a new probe was designed specifically for *L. major *(956 probes). The microarray included a total of 8,978 70-mer probes that recognized with no mismatches all *L. infantum *genes (8,184, GeneDB version 3) and also all *L. major *genes (8,370, GeneDB version 5.1) with a small percentage of the probes having at most 2 mismatches. Also, 372 control probes were included in the microarray for assessing synthesis variability, and location of the probe within a given open reading frame and of mismatches on hybridization. The probes were synthesized in 384-well plates by Invitrogen. The microarrays were printed on SuperChip (Erie Scientific, Portsmouth, NH, USA) using a BioRobotics MicroGrid (Genomic solutions Inc, Ann Arbor, MI, USA). Each probe was printed in duplicate. Our microarray platform is described in the Gene Expression Omnibus (GEO) with accession number GPL6855.

### Total RNA preparation and labeling

Total RNA was isolated from 10^8 ^*Leishmania *cells during the mid-log phase using RNeasy Plus Mini Kit (QIAGEN, Hilden, Germany). The RNA preparation was treated with TURBO DNase (Ambion, Austin, TX, USA) to avoid any genomic contamination. The purity, integrity and quantity of the RNA were assessed on the Agilent 2100 bioanalyzer with the RNA 6000 Nano LabChip reagent set (Agilent Technologies, Santa Clara, CA, USA). For each probe, 10 μg of RNA were converted to aminoallyl-dUTP incorporated cDNA using random hexamers (Roche, Basel, Switzerland) and the SuperScript III RNase H Reverse Transcriptase (Invitrogen). Probes were thereafter coupled to the fluorescent dye Alexa Fluor555 or Alexa Fluor647 (Invitrogen) following the manufacturer's recommendations. Fluorescent probes were then purified with MinElute Spin Columns (QIAGEN) and quantified spectrophotometrically.

### Genomic DNA preparation and labeling

Genomic DNA from 10^8 ^cells was isolated using the DNAzol technique (Invitrogen) as recommended by the manufacturer. Total DNA was then fragmented by successive passages through 22G1" and 27G 1/2" needles (Becton Dickinson Franklin Lakes, NJ, USA). Fragmented DNA was then double digested with *Pvu*II and *Mse*I restriction enzymes. Digested DNA was purified by phenol-chloroform, followed by an ethanol precipitation. For each probe, 4 μg of purified fragmented and digested genomic DNA were converted to fluorescently labeled DNA using Cy5- or Cy3-dCTP (Amersham, Piscataway, NJ, USA), random hexamers (Roche) and the exo^- ^Klenow DNA polymerase (NEB, Ipswich, MA, USA). Fluorescent probes were then purified with ArrayIt columns (TeleChem International, Sunnyvale, CA, USA) and quantified spectrophotometrically.

### Microarray hybridization

Prehybridization and hybridization were performed at 42°C under immersion (Corning chambers, Corning, NY, USA). Slides were prehybridized for 90 minutes in PreHYB Solution (5× Denhardt, 30% formamide, 6× SSPE, 0.5% SDS, 100 μg/ml salmon sperm DNA). Then, slides were first washed 2 times at 42°C for 5 minutes in 2× SSC, 0.1% SDS with gentle agitation. Subsequent washes were at room temperature, 3 minutes each, in 1× SSC, 0.2× SSC and 0.05× SSC. Slides were then dipped in 100% isopropanol and dried by centrifugation. For hybridization, Alexa Fluor555 and 647 cDNA probes were dried and resuspended in the HYB solution (2.5× Denhardt, 30% formamide, 6× SSPE, 0.5% SDS, 100 μg/ml salmon sperm DNA, 750 μg/ml yeast tRNA), then mixed, denatured 5 minutes at 95°C and cooled slowly to 42°C. Mixed probes were applied on the array under a lifterslip. Hybridization was performed for 16 h. Washes after hybridization were the same as those described for the prehybridization.

### Fluorescence detection, data processing and statistical analysis

The Perkin Elmer ScanArray 4000XL Scanner was used for image acquisition (Perkin Elmer, Waltham, MA, USA). GenePix Pro 6.0 image analysis software (Axon Instruments, Union City, CA, USA) was used to quantify the fluorescence signal intensities of the array features. Four different RNA preparations of each mutant and their respective wild-type strain were analyzed, including dye-swaps. Raw data from GenePix were imported in R 2.2.1 for normalization and statistical analyses were performed using the LIMMA (version 2.7.3) package [83-85]. Before processing, probes were flagged according to the hybridization signal quality [86]. Weights were assigned to each array in order to give less weight to arrays of lesser quality [87]. Data were corrected using background subtraction based on convolution of normal and exponential distributions [88]. Intra-array normalization was carried out using the 'print-tip loess' statistical method and inter-array normalization was done by using the 'quantiles of A' method for each array [89]. Statistical analysis was done using linear model fitting and standard errors were moderated using a simple empirical Bayes [83]. Multiple testing corrections were done using the FDR method with a threshold *p*-value of 0.05. Only genes statistically significant with an absolute log ratio greater than 0.58 (log_2 _1.5) were considered as differentially expressed. Species comparison was performed only on probes that had less than two mismatches when hybridized to either *Leishmania *species. GeneSpring GX 3.1 was used for the generation of scatter plots and for chromosome by chromosome analysis. The entire data set has been deposited in GEO under the accession number series GSE9949. The comparative genomic hybridization data are deposited under reference number GSE11623.

### qRT-PCR

Three independent RNA preparations were conducted for each condition. First-strand cDNA was synthesized from 2 μg of total RNA using the Superscript III RNase H Reverse Transcriptase enzyme and random hexamers (Roche) according to the manufacturer's instructions. The resulting cDNA samples were stored at -20°C until use. Control PCR amplification was carried out using primers from different internal controls (*GAPDH *and *actin*) to evaluate the uniformity of cDNA synthesis in different samples. Primers, TaqMan probes, experimental procedures and quantification for qRT-PCR of the folate transporter genes was as described (AA Ouameur *et al*., unpublished data) using the glyceraldehyde-3-phosphate dehydrogenase gene (*GAPDH*) for normalization. For all other genes, equal amounts of cDNA were run in triplicate and amplified in a 15 μl reaction containing 7.5 μl of 2× Universal PCR Master Mix (Applied Biosystems, Foster City, CA, USA), 10 nM of Z-tailed forward primer, 100 nM of reverse primer, 250 nM of Amplifluor Uniprimer probe (Chemicon Int., Temecula, CA, USA), and 1 μl of cDNA target. Reactions were performed at the Gene Quantification core laboratory of the Centre de Génomique de Québec using the Applied Biosystems Prism 7900 Sequence Detector [90]. Amplification was normalized to two genes showing a highly stable expression in wild-type and resistant strains: *LinJ18_V3.0630*/*LmjF18.0620 *encoding a putative 60S ribosomal protein L10a, and *LinJ36_V3.0850/LmjF36.2500 *encoding a chromatin assembly factor 1 subunit b-like protein.

## Abbreviations

CGH, comparative genomic hybridization; DHFR, dihydrofolate reductase; DHFR-TS, DHFR-thymidylate synthase; FBT, folate biopterin transporter; FT, folate transporter; GEO, Gene Expression Omnibus; MTX, methotrexate; PFGE, pulsed-field gel electrophoresis; PTR, pteridine reductase; qRT-PCR, quantitative real-time reverse transcription PCR.

## Authors' contributions

JM carried out the molecular genetic studies and all the microarray hybridizations performed in this study, participated in the bioinformatic analyses of microarray data and drafted the manuscript. AHO helped in the design of qRT-PCR assays. DL developed and optimized the comparative genomic hybridization protocol. PR designed the 70-mer *Leishmania *oligonucleotide microarrays. FR performed the microarray normalization and statistical analysis. SB developed the LIMS that was used to integrate microarray results storage and analysis. JC, MOl, MOu, BP and MJT are part of a CIHR group grant and have supervised all the experiments presented in this paper. All authors read and approved the final manuscript.

## Additional data files

The following additional data are available with the online version of this paper. Additional data file 1 contains Table S1, which lists the differential expression measured by the full-genome microarray analysis. Additional data file 2 contains supplementary Figures S1-S3. Additional data file 3 contains supplementary Figure S4, which shows the results of the comparative genomic hybridization analyses of *L. major *MTX60.4 versus the respective wild-type cells.

## Supplementary Material

Additional data file 1 Differential expression measured by the full-genome microarray analysis. Click here for file

Additional data file 2 Figure S1 shows the direct repeats flanking the *DHFR-TS *locus of *L. major *and *L. infantum *chromosome 6, and also provides the circular junction sequence formed by homologous recombination. Figure S2 shows the inverted repeats present on chromosome 23 of *L. infantum*, and provides the sequence of the new junction formed through the inverted duplication. Figure S3 shows the sequence of the *L. major *chimera gene *LmjF10.0380/0390*. Click here for file

Additional data file 3 Results of the comparative genomic hybridization analyses of *L. major *MTX60.4 versus the respective wild-type cells. Click here for file

## References

[B1] Handman E (2001). *Leishmania *sis: current status of vaccine development.. Clin Microbiol Rev.

[B2] Sundar S, More DK, Singh MK, Singh VP, Sharma S, Makharia A, Kumar PC, Murray HW (2000). Failure of pentavalent antimony in visceral leishmaniasis in India: report from the center of the Indian epidemic.. Clin Infect Dis.

[B3] Beverley SM (1991). Gene amplification in *Leishmania*.. Annu Rev Microbiol.

[B4] Borst P, Ouellette M (1995). New mechanisms of drug resistance in parasitic protozoa.. Annu Rev Microbiol.

[B5] Coderre JA, Beverley SM, Schimke RT, Santi DV (1983). Overproduction of a bifunctional thymidylate synthetase-dihydrofolate reductase and DNA amplification in methotrexate-resistant *Leishmania *tropica.. Proc Natl Acad Sci USA.

[B6] Beverley SM, Coderre JA, Santi DV, Schimke RT (1984). Unstable DNA amplifications in methotrexate-resistant *Leishmania *consist of extrachromosomal circles which relocalize during stabilization.. Cell.

[B7] Garvey EP, Santi DV (1986). Stable amplified DNA in drug-resistant *Leishmania *exists as extrachromosomal circles.. Science.

[B8] Dewes H, Ostergaard HL, Simpson L (1986). Impaired drug uptake in methotrexate resistant *Crithidia fasciculata *without changes in dihydrofolate reductase activity or gene amplification.. Mol Biochem Parasitol.

[B9] Nare B, Luba J, Hardy LW, Beverley S (1997). New approaches to *Leishmania *chemotherapy: pteridine reductase 1 (PTR1) as a target and modulator of antifolate sensitivity [In Process Citation].. Parasitology.

[B10] Ouellette M, Drummelsmith J, El Fadili A, Kundig C, Richard D, Roy G (2002). Pterin transport and metabolism in *Leishmania *and related trypanosomatid parasites.. Int J Parasitol.

[B11] Hardy LW, Matthews W, Nare B, Beverley SM (1997). Biochemical and genetic tests for inhibitors of *Leishmania *pteridine pathways.. Exp Parasitol.

[B12] Chowdhury SF, Di Lucrezia R, Guerrero RH, Brun R, Goodman J, Ruiz-Perez LM, Pacanowska DG, Gilbert IH (2001). Novel inhibitors of *Leishmania *l dihydrofolate reductase.. Bioorg Med Chem Lett.

[B13] Khabnadideh S, Pez D, Musso A, Brun R, Perez LM, Gonzalez-Pacanowska D, Gilbert IH (2005). Design, synthesis and evaluation of 2,4-diaminoquinazolines as inhibitors of trypanosomal and leishmanial dihydrofolate reductase.. Bioorg Med Chem.

[B14] Ellenberger TE, Beverley SM (1987). Reductions in methotrexate and folate influx in methotrexate-resistant lines of *Leishmania major *are independent of R or H region amplification.. J Biol Chem.

[B15] Kaur K, Coons T, Emmett K, Ullman B (1988). Methotrexate-resistant *Leishmania donovani *genetically deficient in the folate-methotrexate transporter.. J Biol Chem.

[B16] Papadopoulou B, Roy G, Ouellette M (1993). Frequent amplification of a short chain dehydrogenase gene as part of circular and linear amplicons in methotrexate resistant *Leishmania*.. Nucleic Acids Res.

[B17] Gamarro F, Chiquero MJ, Amador MV, Legare D, Ouellette M, Castanys S (1994). P-glycoprotein overexpression in methotrexate-resistant *Leishmania tropica*.. Biochem Pharmacol.

[B18] Cunningham ML, Beverley SM (2001). Pteridine salvage throughout the *Leishmania *infectious cycle: implications for antifolate chemotherapy.. Mol Biochem Parasitol.

[B19] Richard D, Leprohon P, Drummelsmith J, Ouellette M (2004). Growth phase regulation of the main folate transporter of *Leishmania infantum *and its role in methotrexate resistance.. J Biol Chem.

[B20] Richard D, Kundig C, Ouellette M (2002). A new type of high affinity folic acid transporter in the protozoan parasite *Leishmania *and deletion of its gene in methotrexate-resistant cells.. J Biol Chem.

[B21] El Fadili A, Kundig C, Roy G, Ouellette M (2004). Inactivation of the *Leishmania tarentolae *pterin transporter (BT1) and reductase (PTR1) genes leads to viable parasites with changes in folate metabolism and hypersensitivity to the antifolate methotrexate.. J Biol Chem.

[B22] Ouellette M, Hettema E, Wust D, Fase-Fowler F, Borst P (1991). Direct and inverted DNA repeats associated with P-glycoprotein gene amplification in drug resistant *Leishmania*.. EMBO J.

[B23] Grondin K, Papadopoulou B, Ouellette M (1993). Homologous recombination between direct repeat sequences yields P-glycoprotein containing amplicons in arsenite resistant *Leishmania*.. Nucleic Acids Res.

[B24] Grondin K, Roy G, Ouellette M (1996). Formation of extrachromosomal circular amplicons with direct or inverted duplications in drug-resistant *Leishmania tarentolae*.. Mol Cell Biol.

[B25] Hightower RC, Wong ML, Ruiz-Perez L, Santi DV (1987). Electron microscopy of amplified DNA forms in antifolate-resistant *Leishmania*.. J Biol Chem.

[B26] Kapler GM, Beverley SM (1989). Transcriptional mapping of the amplified region encoding the dihydrofolate reductase-thymidylate synthase of *Leishmania major *reveals a high density of transcripts, including overlapping and antisense RNAs.. Mol Cell Biol.

[B27] Arrebola R, Olmo A, Reche P, Garvey EP, Santi DV, Ruiz-Perez LM, Gonzalez-Pacanowska D (1994). Isolation and characterization of a mutant dihydrofolate reductase- thymidylate synthase from methotrexate-resistant *Leishmania *cells.. J Biol Chem.

[B28] Kündig C, Leblanc E, Papadopoulou B, Ouellette M (1999). Role of the locus and of the resistance gene on gene amplification frequency in methotrexate resistant *Leishmania tarentolae*.. Nucleic Acids Res.

[B29] Guimond C, Trudel N, Brochu C, Marquis N, El Fadili A, Peytavi R, Briand G, Richard D, Messier N, Papadopoulou B, Corbeil J, Bergeron MG, Légaré D, Ouellette M (2003). Modulation of gene expression in *Leishmania *drug resistant mutants as determined by targeted DNA microarrays.. Nucleic Acids Res.

[B30] Callahan HL, Beverley SM (1992). A member of the aldoketo reductase family confers methotrexate resistance in *Leishmania*.. J Biol Chem.

[B31] Papadopoulou B, Roy G, Ouellette M (1992). A novel antifolate resistance gene on the amplified H circle of *Leishmania*.. EMBO J.

[B32] Nare B, Hardy LW, Beverley SM (1997). The roles of pteridine reductase 1 and dihydrofolate reductase- thymidylate synthase in pteridine metabolism in the protozoan parasite *Leishmania major*.. J Biol Chem.

[B33] Wang J, Leblanc E, Chang CF, Papadopoulou B, Bray T, Whiteley JM, Lin SX, Ouellette M (1997). Pterin and folate reduction by the *Leishmania tarentolae *H locus short- chain dehydrogenase/reductase PTR1.. Arch Biochem Biophys.

[B34] White TC, Fase-Fowler F, van Luenen H, Calafat J, Borst P (1988). The H circles of *Leishmania tarentolae *are a unique amplifiable system of oligomeric DNAs associated with drug resistance.. J Biol Chem.

[B35] Petrillo-Peixoto ML, Beverley SM (1988). Amplified DNAs in laboratory stocks of *Leishmania tarentolae*: extrachromosomal circles structurally and functionally similar to the inverted-H-region amplification of methotrexate-resistant *Leishmania major*.. Mol Cell Biol.

[B36] Hightower RC, Ruiz-Perez LM, Wong ML, Santi DV (1988). Extrachromosomal elements in the lower eukaryote *Leishmania*.. J Biol Chem.

[B37] Olmo A, Arrebola R, Bernier V, Gonzalez-Pacanowska D, Ruiz-Perez LM (1995). Co-existence of circular and multiple linear amplicons in methotrexate-resistant *Leishmania*.. Nucleic Acids Res.

[B38] Grondin K, Kundig C, Roy G, Ouellette M (1998). Linear amplicons as precursors of amplified circles in methotrexate-resistant *Leishmania tarentolae*.. Nucleic Acids Res.

[B39] El Fadili A, Richard D, Kundig C, Ouellette M (2003). Effect of polyglutamylation of methotrexate on its accumulation and the development of resistance in the protozoan parasite *Leishmania*.. Biochem Pharmacol.

[B40] Gagnon D, Foucher A, Girard I, Ouellette M (2006). Stage specific gene expression and cellular localization of two isoforms of the serine hydroxymethyltransferase in the protozoan parasite *Leishmania*.. Mol Biochem Parasitol.

[B41] Drummelsmith J, Brochu V, Girard I, Messier N, Ouellette M (2003). Proteome mapping of the protozoan parasite *Leishmania *and application to the study of drug targets and resistance mechanisms.. Mol Cell Proteomics.

[B42] Gallego C, Estevez AM, Farez E, Ruiz-Perez LM, Gonzalez-Pacanowska D (2005). Overexpression of AP endonuclease protects *Leishmania major *cells against methotrexate induced DNA fragmentation and hydrogen peroxide.. Mol Biochem Parasitol.

[B43] Wilson M, DeRisi J, Kristensen HH, Imboden P, Rane S, Brown PO, Schoolnik GK (1999). Exploring drug-induced alterations in gene expression in *Mycobacterium tuberculosis *by microarray hybridization.. Proc Natl Acad Sci USA.

[B44] Lee CH, Macgregor PF (2004). Using microarrays to predict resistance to chemotherapy in cancer patients.. Pharmacogenomics.

[B45] Brazas MD, Hancock RE (2005). Using microarray gene signatures to elucidate mechanisms of antibiotic action and resistance.. Drug Discov Today.

[B46] Ouellette M, Drummelsmith J, Leprohon P, El Fadili K, Foucher A, Vergnes B, Légaré D (2007). Drug Resistance in Leishmania.

[B47] Saxena A, Worthey EA, Yan S, Leland A, Stuart KD, Myler PJ (2003). Evaluation of differential gene expression in *Leishmania major *Friedlin procyclics and metacyclics using DNA microarray analysis.. Mol Biochem Parasitol.

[B48] Akopyants NS, Matlib RS, Bukanova EN, Smeds MR, Brownstein BH, Stormo GD, Beverley SM (2004). Expression profiling using random genomic DNA microarrays identifies differentially expressed genes associated with three major developmental stages of the protozoan parasite *Leishmania major*.. Mol Biochem Parasitol.

[B49] Saxena A, Lahav T, Holland N, Aggarwal G, Anupama A, Huang Y, Volpin H, Myler PJ, Zilberstein D (2007). Analysis of the *Leishmania donovani *transcriptome reveals an ordered progression of transient and permanent changes in gene expression during differentiation.. Mol Biochem Parasitol.

[B50] Srividya G, Duncan R, Sharma P, Raju BV, Nakhasi HL, Salotra P (2007). Transcriptome analysis during the process of *in vitro *differentiation of *Leishmania donovani *using genomic microarrays.. Parasitology.

[B51] Almeida R, Gilmartin BJ, McCann SH, Norrish A, Ivens AC, Lawson D, Levick MP, Smith DF, Dyall SD, Vetrie D, Freeman TC, Coulson RM, Sampaio I, Schneider H, Blackwell JM (2004). Expression profiling of the *Leishmania *life cycle: cDNA arrays identify developmentally regulated genes present but not annotated in the genome.. Mol Biochem Parasitol.

[B52] Singh N, Almeida R, Kothari H, Kumar P, Mandal G, Chatterjee M, Venkatachalam S, Govind MK, Mandal SK, Sundar S (2007). Differential gene expression analysis in antimony-unresponsive Indian kala azar (visceral leishmaniasis) clinical isolates by DNA microarray.. Parasitology.

[B53] McNicoll F, Drummelsmith J, Muller M, Madore E, Boilard N, Ouellette M, Papadopoulou B (2006). A combined proteomic and transcriptomic approach to the study of stage differentiation in *Leishmania infantum*.. Proteomics.

[B54] Leprohon P, Legare D, Girard I, Papadopoulou B, Ouellette M (2006). Modulation of *Leishmania *ABC protein gene expression through life stages and among drug-resistant parasites.. Eukaryot Cell.

[B55] Holzer TR, McMaster WR, Forney JD (2006). Expression profiling by whole-genome interspecies microarray hybridization reveals differential gene expression in procyclic promastigotes, lesion-derived amastigotes, and axenic amastigotes in *Leishmania mexicana*.. Mol Biochem Parasitol.

[B56] Leifso K, Cohen-Freue G, Dogra N, Murray A, McMaster WR (2007). Genomic and proteomic expression analysis of *Leishmania *promastigote and amastigote life stages: the *Leishmania *genome is constitutively expressed.. Mol Biochem Parasitol.

[B57] El Fadili K, Messier N, Leprohon P, Roy G, Guimond C, Trudel N, Saravia NG, Papadopoulou B, Legare D, Ouellette M (2005). Role of the ABC transporter MRPA (PGPA) in antimony resistance in *Leishmania infantum *axenic and intracellular amastigotes.. Antimicrob Agents Chemother.

[B58] Clayton CE (2002). Life without transcriptional control? From fly to man and back again.. EMBO J.

[B59] Blaineau C, Bastien P, Rioux JA, Roizes G, Pages M (1991). Long-range restriction maps of size-variable homologous chromosomes in *Leishmania infantum*.. Mol Biochem Parasitol.

[B60] Sanger Institute Pathogen Sequencing Unit. http://www.genedb.org/.

[B61] Duesberg P, Li R, Sachs R, Fabarius A, Upender MB, Hehlmann R (2007). Cancer drug resistance: the central role of the karyotype.. Drug Resist Updat.

[B62] Selmecki A, Forche A, Berman J (2006). Aneuploidy and isochromosome formation in drug-resistant *Candida albicans*.. Science.

[B63] Coste A, Selmecki A, Forche A, Diogo D, Bougnoux ME, d'Enfert C, Berman J, Sanglard D (2007). Genotypic evolution of azole resistance mechanisms in sequential *Candida albicans *isolates.. Eukaryot Cell.

[B64] Haimeur A, Guimond C, Pilote S, Mukhopadhyay R, Rosen BP, Poulin R, Ouellette M (1999). Elevated levels of polyamines and trypanothione resulting from overexpression of the ornithine decarboxylase gene in arsenite-resistant *Leishmania*.. Mol Microbiol.

[B65] Drummelsmith J, Girard I, Trudel N, Ouellette M (2004). Differential protein expression analysis of *Leishmania major *reveals novel roles for methionine adenosyltransferase and S-adenosylmethionine in methotrexate resistance.. J Biol Chem.

[B66] Marquis N, Gourbal B, Rosen BP, Mukhopadhyay R, Ouellette M (2005). Modulation in aquaglyceroporin AQP1 gene transcript levels in drug-resistant *Leishmania*.. Mol Microbiol.

[B67] Ibrahim ME, Barker DC (2001). The origin and evolution of the *Leishmania donovani *complex as inferred from a mitochondrial cytochrome oxidase II gene sequence.. Infect Genet Evol.

[B68] Rovai L, Tripp C, Stuart K, Simpson L (1992). Recurrent polymorphisms in small chromosomes of *Leishmania tarentolae *after nutrient stress or subcloning.. Mol Biochem Parasitol.

[B69] Tripp CA, Myler PJ, Stuart K (1991). A DNA sequence (LD1) which occurs in several genomic organizations in *Leishmania*.. Mol Biochem Parasitol.

[B70] Navarro M, Liu J, Muthui D, Ortiz G, Segovia M, Hamers R (1994). Inverted repeat structure and homologous sequences in the LD1 amplicons of *Leishmania *spp.. Mol Biochem Parasitol.

[B71] Ortiz G, Segovia M (1996). Characterisation of the novel junctions of two minichromosomes of *Leishmania major*.. Mol Biochem Parasitol.

[B72] Dubessay P, Ravel C, Bastien P, Lignon MF, Ullman B, Pages M, Blaineau C (2001). Effect of large targeted deletions on the mitotic stability of an extra chromosome mediating drug resistance in *Leishmania*.. Nucleic Acids Res.

[B73] Genest PA, ter Riet B, Dumas C, Papadopoulou B, van Luenen HG, Borst P (2005). Formation of linear inverted repeat amplicons following targeting of an essential gene in *Leishmania*.. Nucleic Acids Res.

[B74] Butler DK, Yasuda LE, Yao MC (1995). An intramolecular recombination mechanism for the formation of the rRNA gene palindrome of *Tetrahymena thermophila*.. Mol Cell Biol.

[B75] Albrecht EB, Hunyady AB, Stark GR, Patterson TE (2000). Mechanisms of sod2 gene amplification in *Schizosaccharomyces pombe*.. Mol Biol Cell.

[B76] Tanaka H, Tapscott SJ, Trask BJ, Yao MC (2002). Short inverted repeats initiate gene amplification through the formation of a large DNA palindrome in mammalian cells.. Proc Natl Acad Sci USA.

[B77] Tanaka H, Cao Y, Bergstrom DA, Kooperberg C, Tapscott SJ, Yao MC (2007). Intrastrand annealing leads to the formation of a large DNA palindrome and determines the boundaries of genomic amplification in human cancer.. Mol Cell Biol.

[B78] Cruz AK, Titus R, Beverley SM (1993). Plasticity in chromosome number and testing of essential genes in *Leishmania *by targeting.. Proc Natl Acad Sci USA.

[B79] Tanaka H, Bergstrom DA, Yao MC, Tapscott SJ (2005). Widespread and nonrandom distribution of DNA palindromes in cancer cells provides a structural platform for subsequent gene amplification.. Nat Genet.

[B80] Sambrook J, Fritsch EF, Maniatis T (1989). Molecular Cloning.

[B81] Ivens AC, Peacock CS, Worthey EA, Murphy L, Aggarwal G, Berriman M, Sisk E, Rajandream MA, Adlem E, Aert R, Anupama A, Apostolou Z, Attipoe P, Bason N, Bauser C, Beck A, Beverley SM, Bianchettin G, Borzym K, Bothe G, Bruschi CV, Collins M, Cadag E, Ciarloni L, Clayton C, Coulson RM, Cronin A, Cruz AK, Davies RM, De Gaudenzi J (2005). The genome of the kinetoplastid parasite, *Leishmania major*.. Science.

[B82] Peacock CS, Seeger K, Harris D, Murphy L, Ruiz JC, Quail MA, Peters N, Adlem E, Tivey A, Aslett M, Kerhornou A, Ivens A, Fraser A, Rajandream MA, Carver T, Norbertczak H, Chillingworth T, Hance Z, Jagels K, Moule S, Ormond D, Rutter S, Squares R, Whitehead S, Rabbinowitsch E, Arrowsmith C, White B, Thurston S, Bringaud F, Baldauf SL (2007). Comparative genomic analysis of three *Leishmania *species that cause diverse human disease.. Nat Genet.

[B83] Smyth GK (2004). Linear models and empirical bayes methods for assessing differential expression in microarray experiments.. Stat Appl Genet Mol Biol.

[B84] Smyth GK, Speed T (2003). Normalization of cDNA microarray data.. Methods.

[B85] Smyth GK, Michaud J, Scott HS (2005). Use of within-array replicate spots for assessing differential expression in microarray experiments.. Bioinformatics.

[B86] Oshlack A, Chabot AE, Smyth GK, Gilad Y (2007). Using DNA microarrays to study gene expression in closely related species.. Bioinformatics.

[B87] Ritchie ME, Diyagama D, Neilson J, van Laar R, Dobrovic A, Holloway A, Smyth GK (2006). Empirical array quality weights in the analysis of microarray data.. BMC Bioinformatics.

[B88] Ritchie ME, Silver J, Oshlack A, Holmes M, Diyagama D, Holloway A, Smyth GK (2007). A comparison of background correction methods for two-colour microarrays.. Bioinformatics.

[B89] Yang Y, Hoh J, Broger C, Neeb M, Edington J, Lindpaintner K, Ott J (2003). Statistical methods for analyzing microarray feature data with replications.. J Comput Biol.

[B90] Quebec Genomics Center. https://genome.ulaval.ca/qrtpcr.

