# Supplementary material for: Modulation of gene expression in drug resistant Leishmania is associated with gene amplification, gene deletion and chromosome aneuploidy
Source: Genome Biol. 2008 Jul 18;9(7):R115. doi: 10.1186/gb-2008-9-7-r115 (PMC2530873; doi:10.1186/gb-2008-9-7-r115)
Supplement: Additional data file 1 — Differential expression measured by the full-genome microarray analysis. [file gb-2008-9-7-r115-S1.doc]

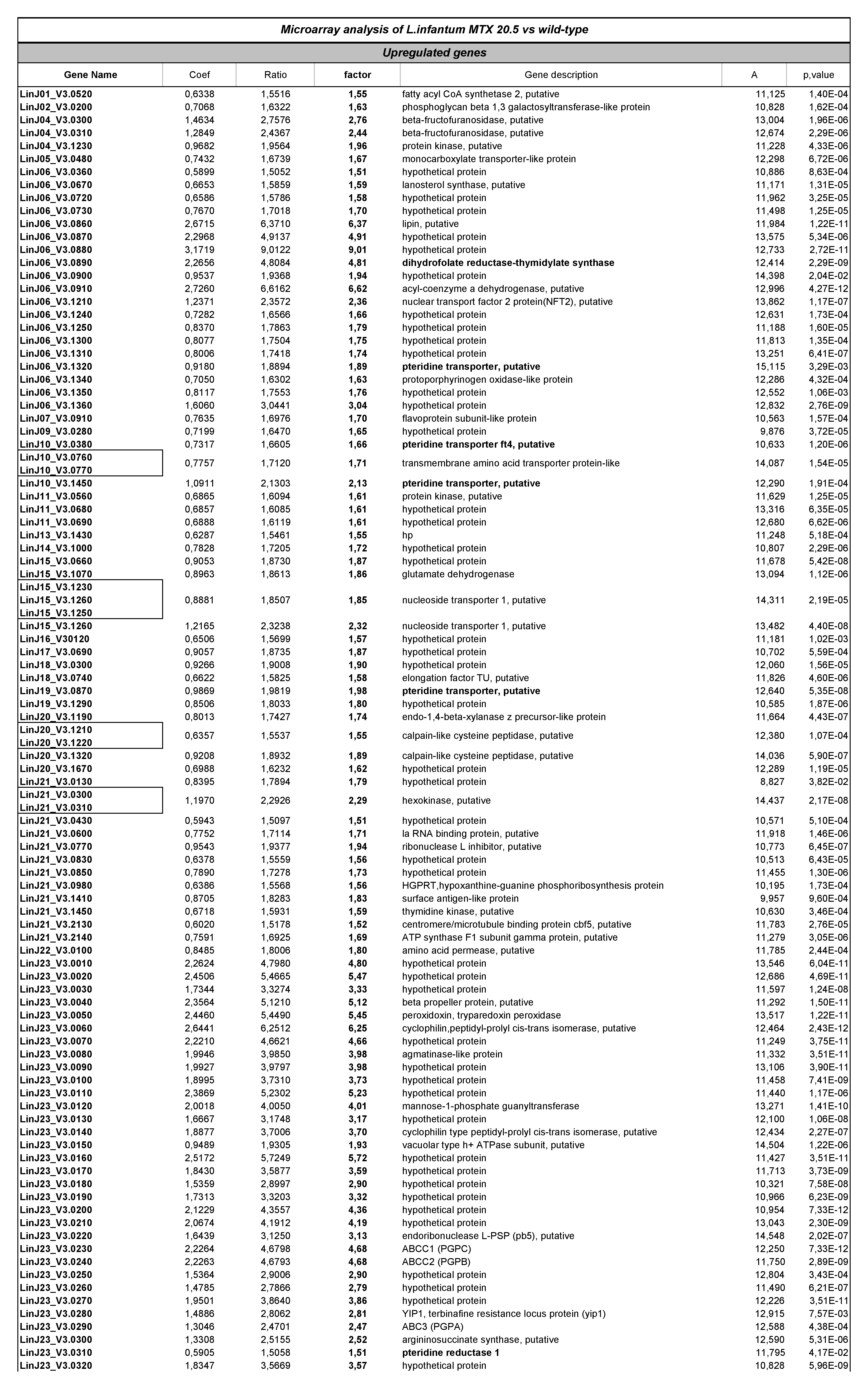


**Table 1A**


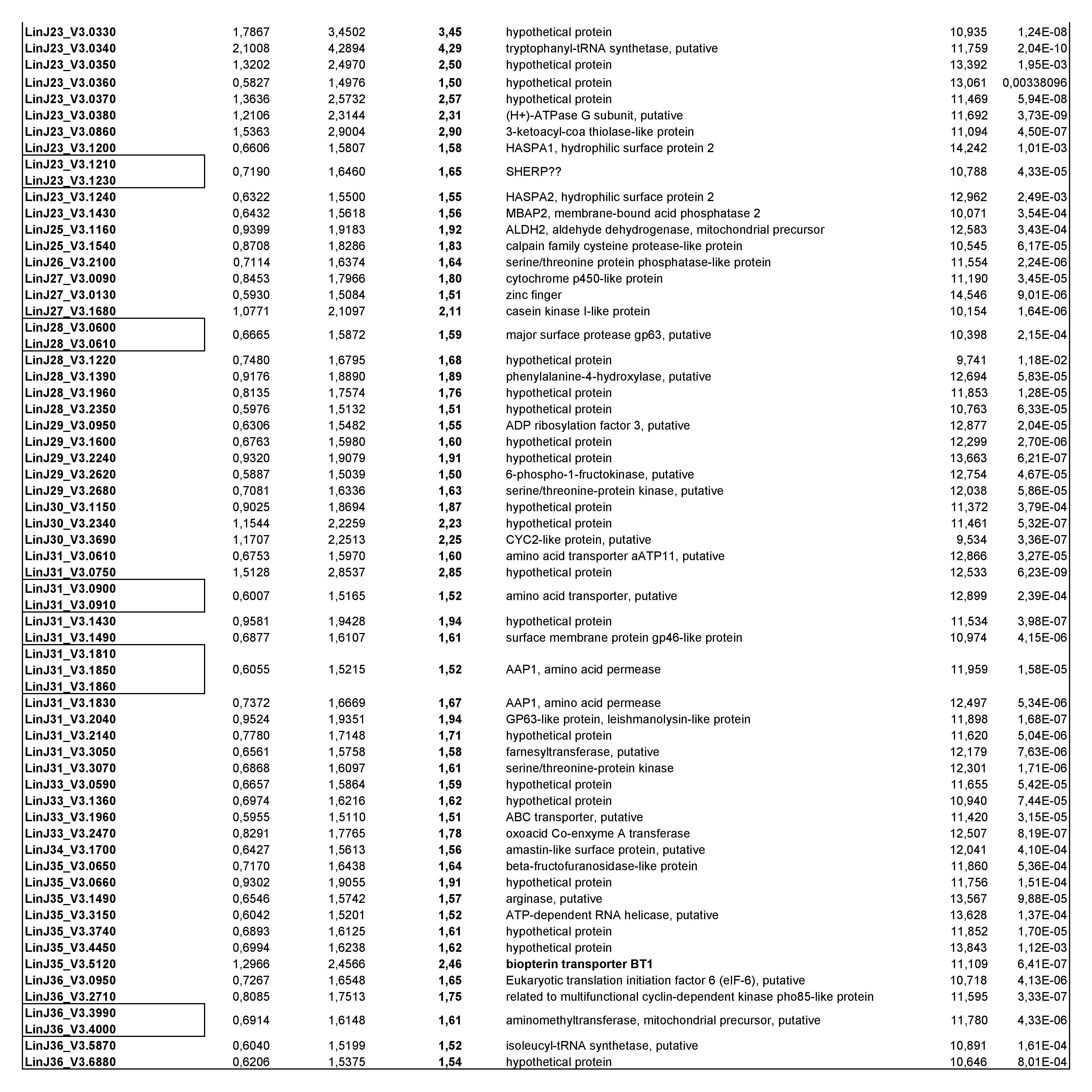


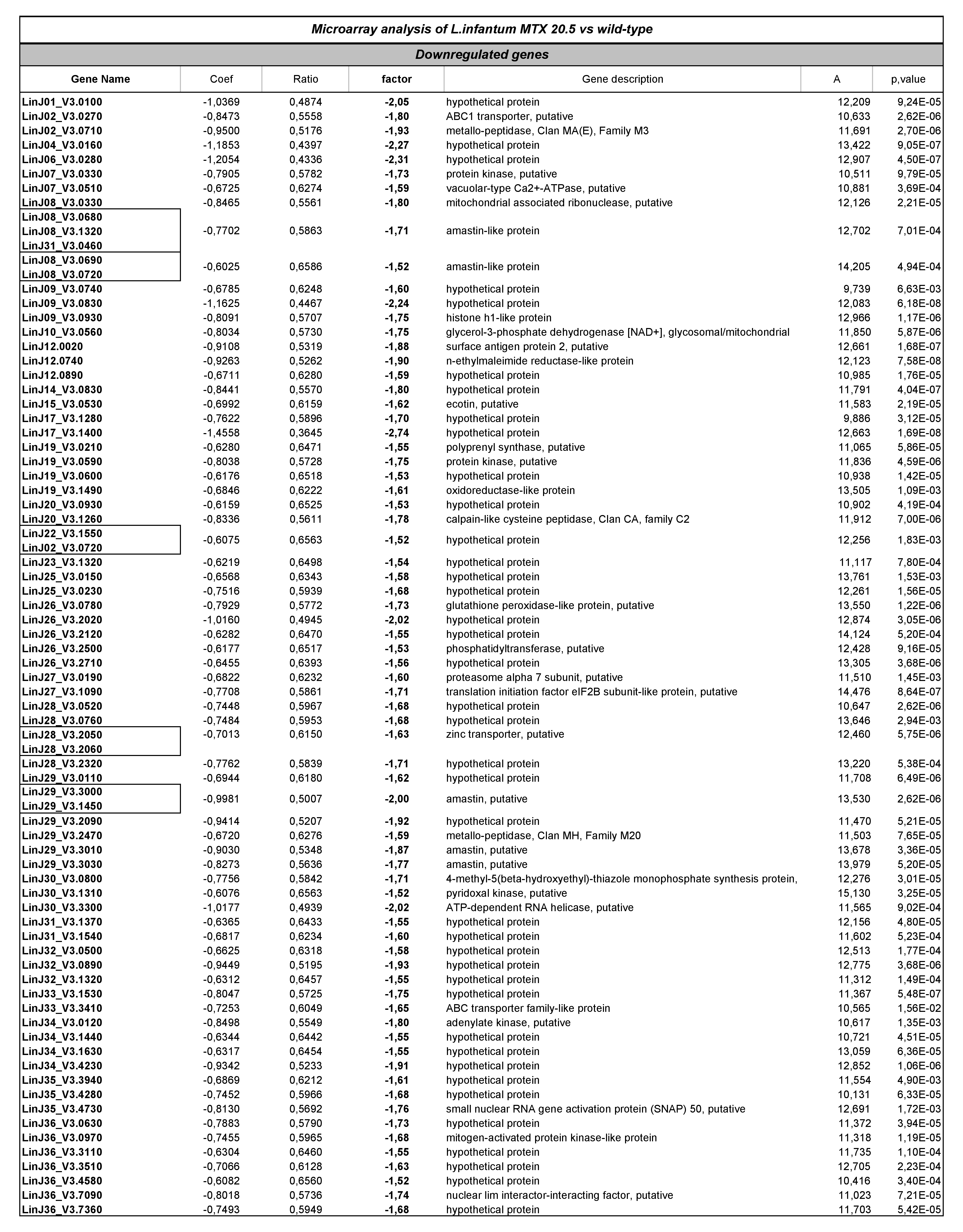


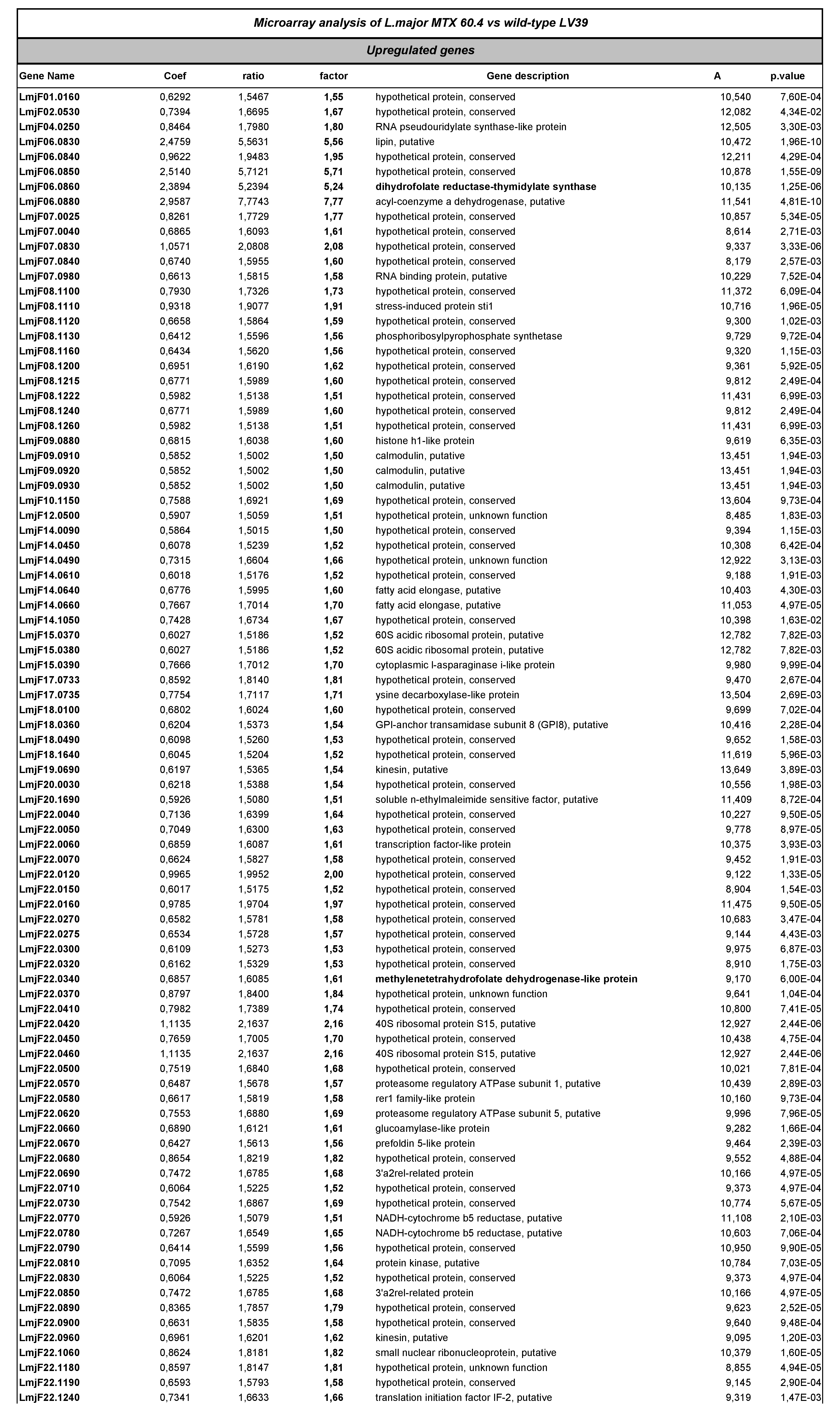


**B**


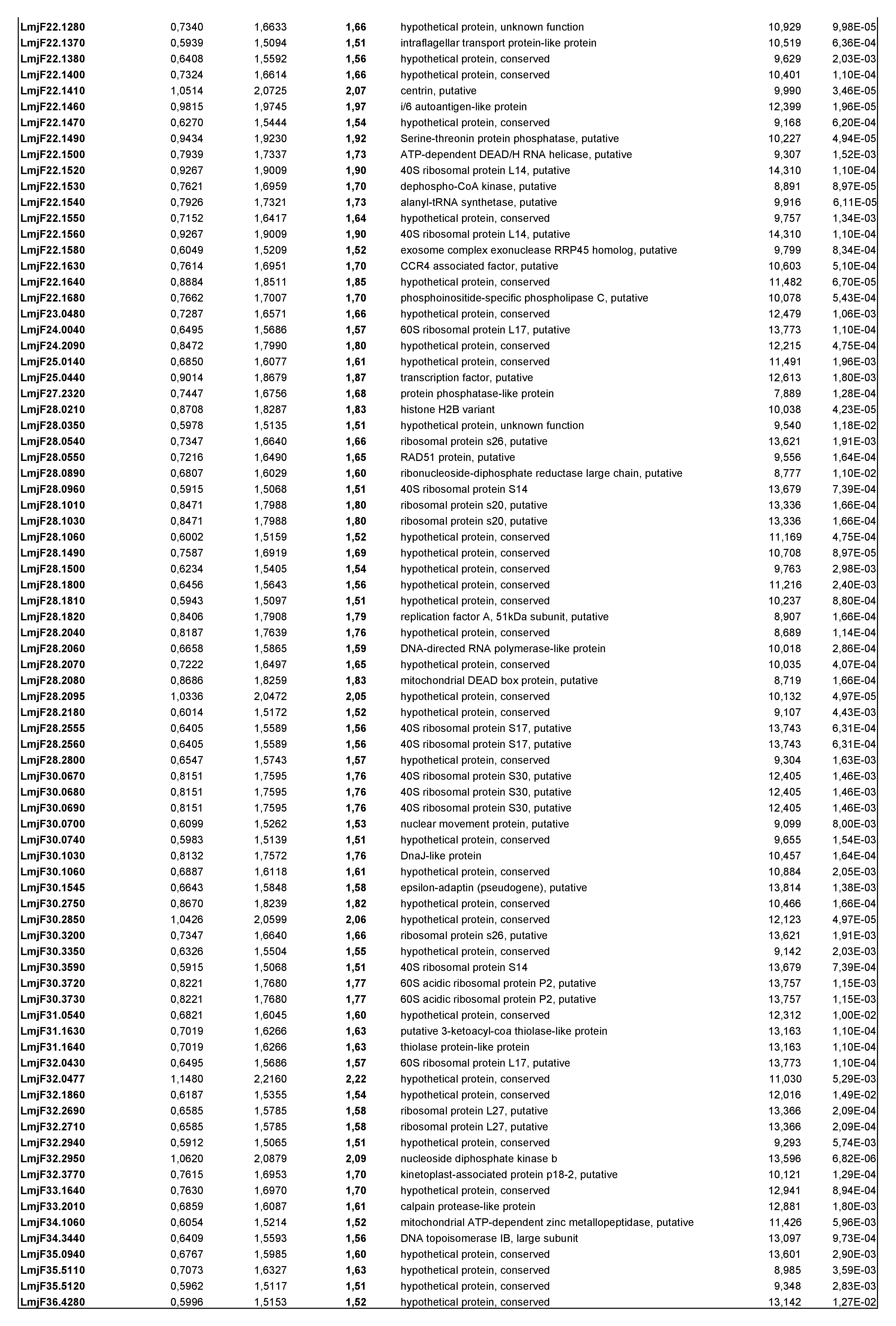


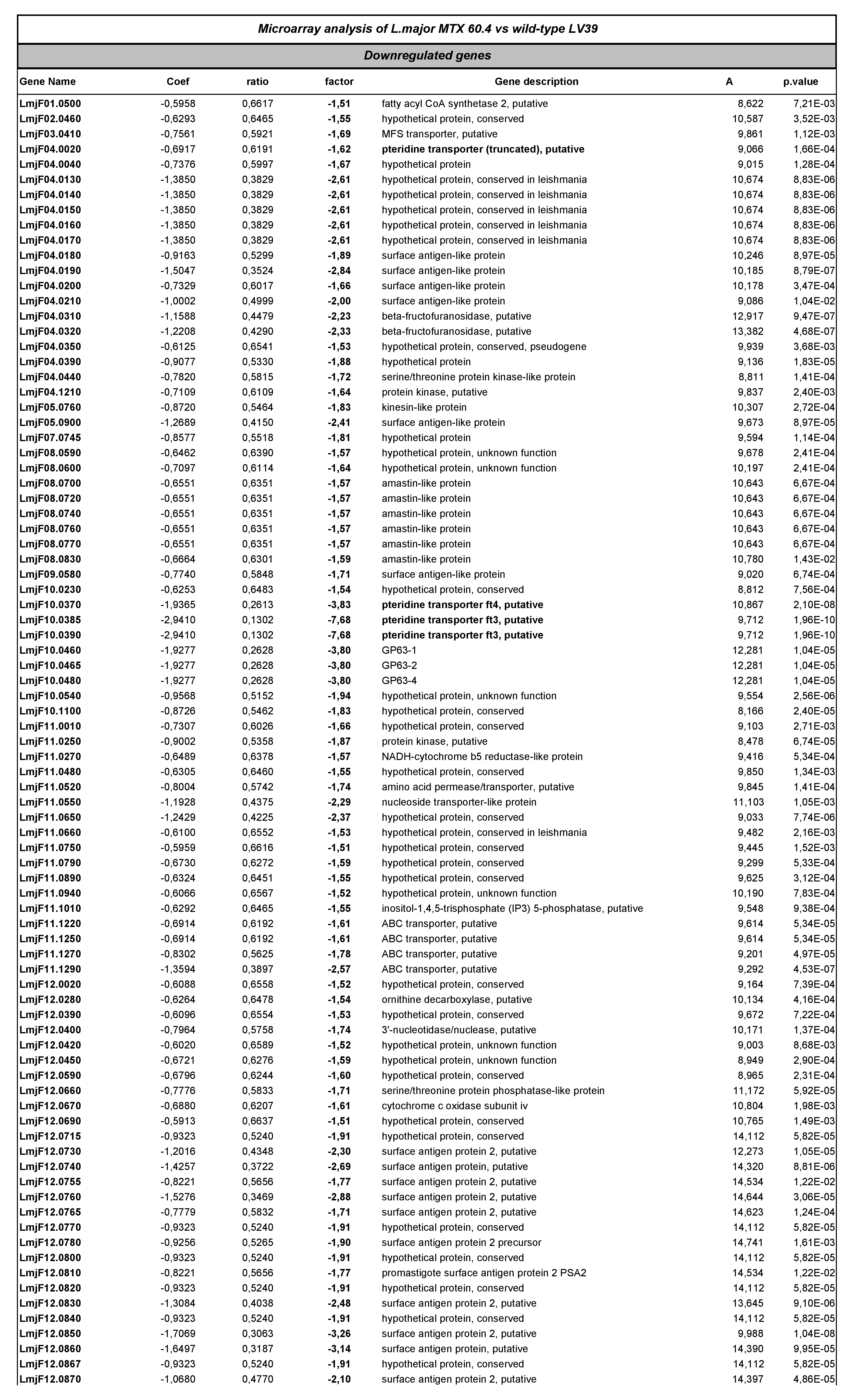


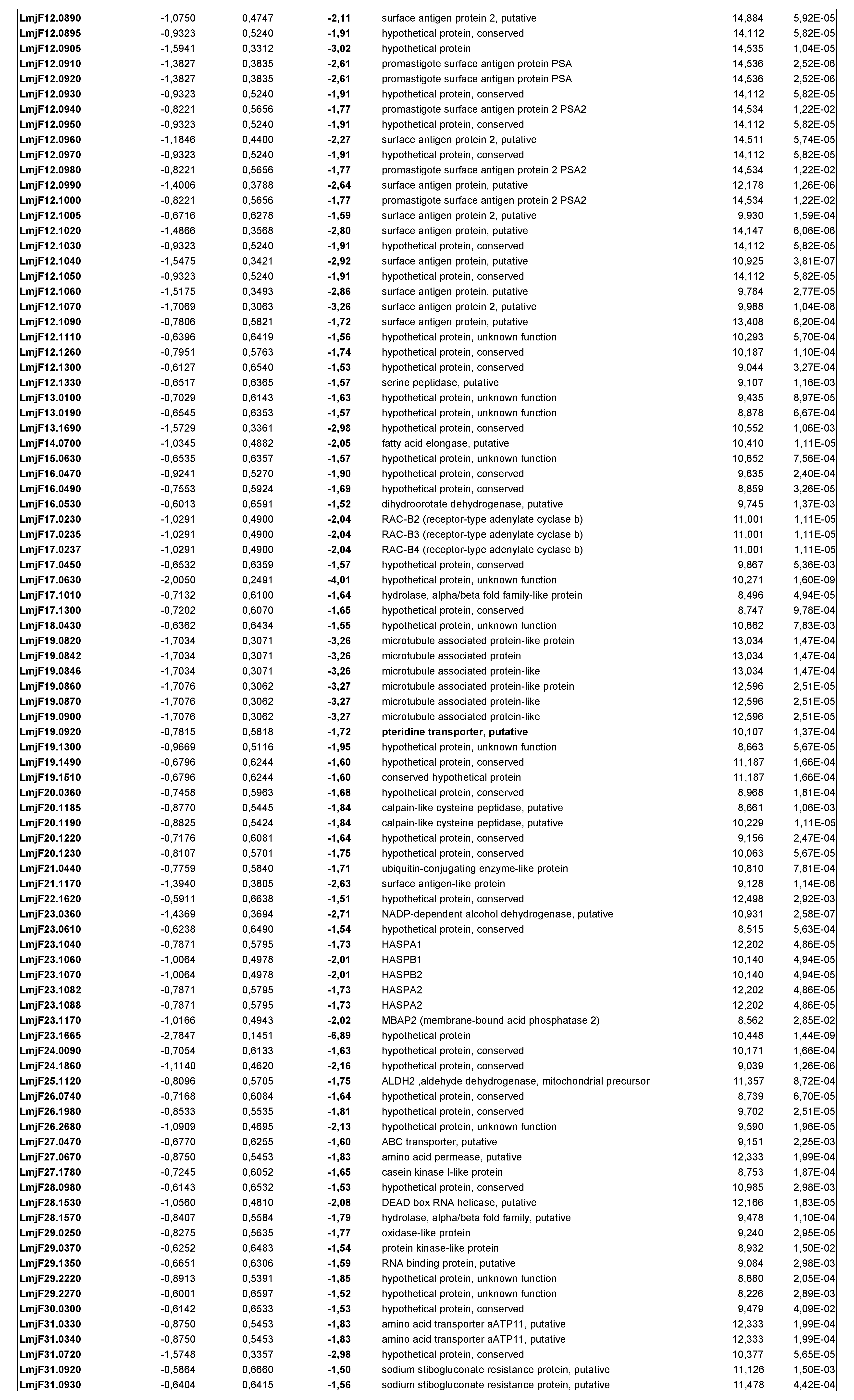


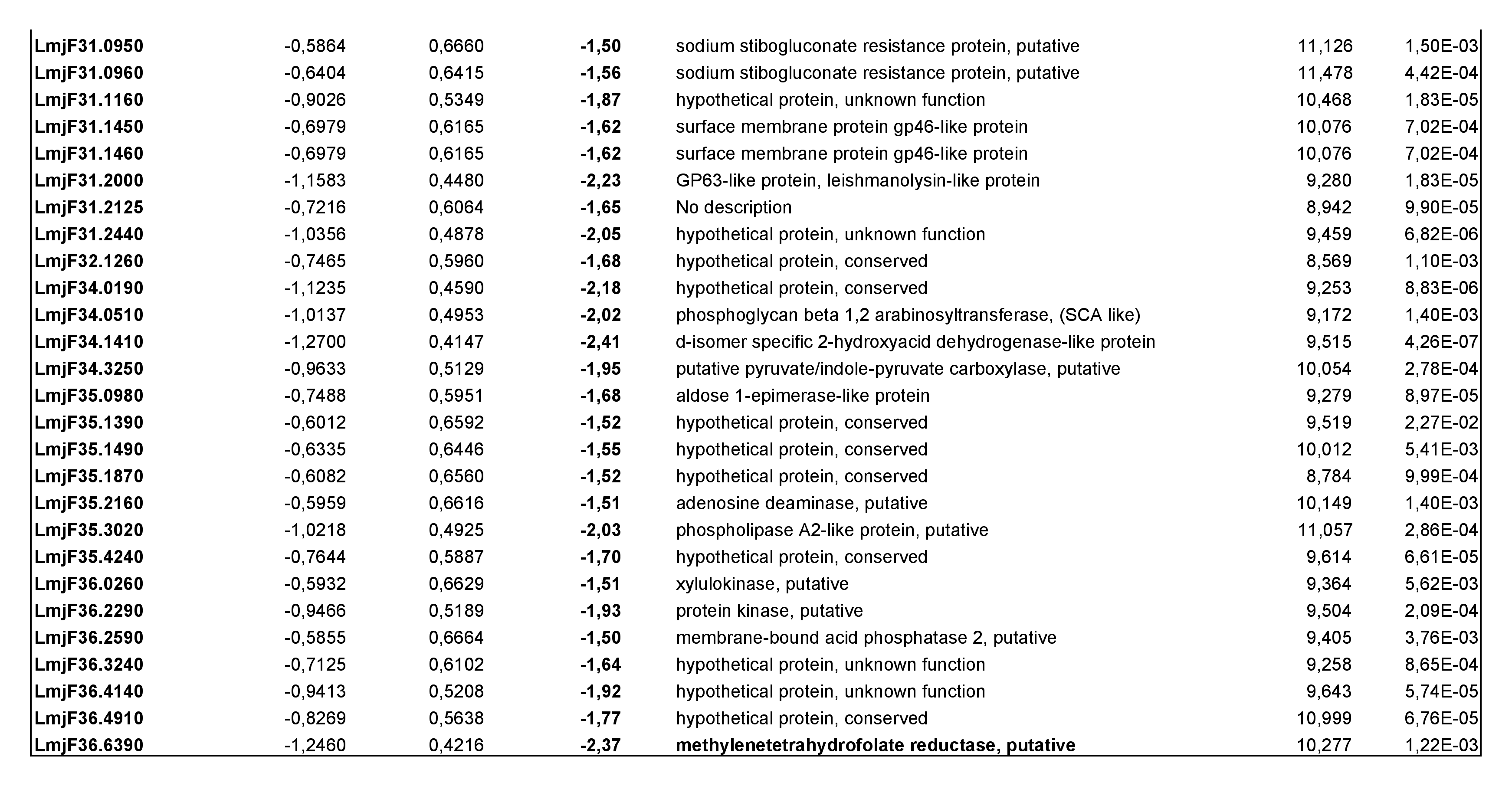


**Table S1** : Differential expression measured by full-genome microarray analysis of (**A**) *L. infantum* MTX20.5 vs *L. infantum* wild-type and (**B**) *L. major* MTX60.4 vs *L. major LV39* wild-type. Gene Name and Gene description columns are according to GeneDB version 3.0 of *L. infantum* genome (A) and version 5.1 of *L. major* genome (B). coef: normalized log_2_ expression ratio (MTX-resistant/wild-type). ratio: normalized expression ratio (MTX-resistant/wild-type). factor: modulated expression factor corresponding to the expression ratio for up-regulated genes, and to the inverse of the expression ratio (-1/ratio) for down-regulated genes. A: results of the inter-array normalization test for significance using the “quantile of A” method (Yang et al. 2003). p.value: related *p-value* calculated with the FDR method, only genes with a *p-value* < 0.05 were considered for analysis. Genes enclosed in boxes represent similar genes that are not discriminated by the array probe.
