# Supplementary material for: Modulation of gene expression in drug resistant Leishmania is associated with gene amplification, gene deletion and chromosome aneuploidy
Source: Genome Biol. 2008 Jul 18;9(7):R115. doi: 10.1186/gb-2008-9-7-r115 (PMC2530873; doi:10.1186/gb-2008-9-7-r115)
Supplement: Additional data file 2 — Figure S1 shows the direct repeats flanking the DHFR-TS locus of L. major and L. infantum chromosome 6, and also provides the circular junction sequence formed by homologous recombination. Figure S2 shows the inverted repeats present on chromosome 23 of L. infantum, and provides the sequence of the new junction formed through the inverted duplication. Figure S3 shows the sequence of the L. major chimera gene LmjF10.0380/0390. [file gb-2008-9-7-r115-S2.doc]

**Fig. S1A. Chromosome 6 repeated sequences in *L. major***

**Seq. 1**: LmjF06_01_20050601_V5.2 (Subsequence range: 343046-367401)

**Seq. 2**: LmjF06_01_20050601_V5.2 (Subsequence range: 369673-390923)

**Identities = 574/575 (99,8%)**

**Seq. 1** 9261 GCTGCCGACGAAGTATCCCCCGCACGCTTGCCCCCTCCGTCTGCTGAGCCAGCGCGTGAGTGCGCACCGGGCCCC 9335

**Seq. 2** 11950 GCTGCCGACGAAGTATCCCCCGCACGCTTGCCCCCTCCGTCTGCTGAGCCAGCGCGTGAGTGCGCACCGGGCCCC 12024

**Seq. 1** 9336 GGCGGCTGACATGTTTTTGGTGCCGTTCTCCTGCGCGTTCACGGAATGTCTTGCGGTTGCACGCGTTTCCGTGGG 9410

**Seq. 2** 12025 GGCGGCTGACATGTTTTTGGTGCCGTTCTCCTGCGCGTTCACGGAATGTCTTGCGGTTGCACGCGTTTCCGTGGG 12099

**Seq. 1** 9411 CGCAACACGGCGGCGAGATCGGGTGGGCTGACCGATGGCGTCCTCGTTTCTTCGCCTCCGTGGAGGTGCGACGTC 9485

**Seq. 2** 12095 CGCAACACGGCGGCGAGATCGGGTGGGCTGACCGATGGCGTCCTCGTTTCTTCGCCTCCGTGGAGGTGCGACGTC 12174

**Seq. 1** 9486 TGTCCCGCCCCTCAGCGACACGTGTGGCCTTCTACTCTCTGTGGGCACGGCCCCCTCCCTTCTCCCTCGTCATCT 9560

**Seq. 2** 12175 TGTCCCGCCCCTCAGCGACACGTGTGGCCTTCTACTCTCTGTGGGCACGGCCCCCTCCCTTCTCCCTCGTCATCT 12249

**Seq. 1** 9561 GCATGGTCCGTGCCTTCAGAGGGCGTCCGACCACGCCCCTCGTGTCTCCGCTTGGTGGGCAGGTAGCGGGCAGGA 9635

**Seq. 2** 12250 GCATGGTCCGTGCCTTCAGAGGGCGTCCGACCACGCCCCTCGTGTCTCCGCTTGGTGGGCAGGTAGCGGGCAGGA 12324

**Seq. 1** 9636 CGAAGCGGGGGCGATGTCCCACACGCCCACGCACCGCCCAGAAGTCGGATGCCTTGCGACGAATCTGCGGACTCG 9710

**Seq. 2** 12325 CGAAGCGGGGGCGATGTCCCACACGCCCACGCACCGCCCAGAAGTCGGATGCCTTGCGACGAATCTGCGGACTCG 12399

**Seq. 1** 9711 CTCCGTGGCCTCGCGCTACCATGCACGCCGTCTCTGCCTCTCCACCGCCACTCTCTCTCTCTCTCTCTCTTCTCC 9785

**Seq. 2** 12400 CTCCGTGGCCTCGCGCTGCCATGCACGCCGTCTCTGCCTCTCCACCGCCACTCTCTCTCTCTCTCTCTCTTCTCC 12474

**Seq. 1** 9786 CTCACACGCTGCTGAAGGAGCGAAGGTACGCGCATGCGCACTGGCCTTGT 9835

**Seq. 2** 12475 CTCACACGCTGCTGAAGGAGCGAAGGTACGCGCATGCGCACTGGCCTTGT 12524

**Fig. S1B. Chromosome 6 repeated sequences in *L. infantum***

**Seq. 3**: LinJ06_20050901_V2.0 (Subsequence range: 349506-373675)

**Seq. 4**: LinJ06_20050901_V2.0 (Subsequence range: 375983-397233)

Identities = 834/837 (99%), Gaps = 0/837 (0%)

**Seq. 3** 2500 TGCCGACGAAGTATCGCCCACCCGCTTGCCCCCTCCTTCTGCTGAACCAGCGCGTGAGTGCGCACCGGGCCCCGGC 2575

**Seq. 4** 3443 TGCCGACGAAGTATCGCCCACCCGCTTGCCCCCTCCTTCTGCTGAACCAGCGCGTGAGTGCGCACCGGGCCCCGGC 3518

**Seq. 3** 2576 GGCCGACATGTTTTGGTGTCGTTCTCTTGCGCGTTCTTTGAATGTTTTGCGGTTGCACGCGTTTCCGTGGACGCAA 2651

**Seq. 4** 3519 GGCCGACATGTTTTGGTGTCGTTCTCTTGCGCGTTCTTTGAATGTTTTGCGGTTGCACGCGTTTCCGTGGACGCAA 3594

**Seq. 3** 2652 CACGGCGACGAGATCAGGTGGACTGACTGATGGCGTCCTCGTTTCTTCGCCTCAGTGGAGGCGTGACGTCTGTCCC 2727

**Seq. 4** 3595 CACGGCGACGAGATCAGGTGGACTGACTGATGGCGTCCTCGTTTCTTCGCCTCAGTGGAGGCGTGACGTCTGTCCC 3670

**Seq. 3** 2728 ACCTCTCAGCGATACGTGTGTGGTCTTCTACTCTCTGTGGGCACGGGCCCCCCGGTGACCGCATGGCCCCCCTTCT 2803

**Seq. 4** 3671 ACCTCTCAGCGATACGTGTGTGGTCTTCTACTCTCTGTGGGCACGGGCCCCCCGGTGACCGCATGGCCCCCCTTCT 3746

**Seq. 3** 2804 TCCTCGTCATCTGCATGGCCCGTGCCTTCAGAGGGCGTCCGGCCACGCCCTTCGTGTCTCCGCTCGGTGGACAGGT 2879

**Seq. 4** 3747 TCCTCGTCATCTGCATGGCCCGTGCCTTCAGAGGGCGTCCGGCCACGCCCTTCGTGTCTCCGCTCGGTGGACAGGT 3822

**Seq. 3** 2880 AGCGGGAAGGACGAAGCGGGTGCGATGGCACACACGCCCACACACCGCCCAGAAGTCGGATGCCTTACGACGAATG 2955

**Seq. 4** 3823 AGCGGGAAGGACGAAGCGGGTGCGATGGCACACACGCCCACACACCGCCCAGAAGTCGGATGCCTTACGACGAATG 3898

**Seq. 3** 2956 TGCTGACTCGCTCCGTGGCCTCGCGCTGCCACGCACTCCGTCTCTGCCTCTCCACCGCCGCTCTCTCTCTTCTCCC 3031

**Seq. 4** 3899 TGCTGACTCGCTCCGTGGCCTCGCGCTGCCACGCACTCCGTCTCTGCCTCTCCACCGCCGCTCTCTCTCTTCTCCC 3974

**Seq. 3** 3032 ACACACGCTGGCGAAGGAGCGAAGGTACTCGCATGCATACCGGCCTTGTTACACACACACACACGCACGCACATCT 3107

**Seq. 4** 3975 ACACACGCTGGCGAAGGAGCGAAGGGACTCGCATGCATACCGGGCTTGGTACACACACACACACGCACGCACATCT 4050

**Seq. 3** 3108 TCGAAACCGCATACATACACAGACACGTACAGCGCACGTGCACGCAGACGTACATCGATCGCGCGCACATCTGCCG 3183

**Seq. 4** 4051 TCGAAACCGCATACATACACAGACACGTACAGCGCACGTGCACGCAGACGTACATCGATCGCGCGCACATCTGCCG 4126

**Seq. 3** 3184 TTGCACACACTAACCGCTTTCCTCGTAAATCTCACGGACACGCGTGCGTGCACCTATCCGCCTCACCCTGCACTTT 3259

**Seq. 4** 4127 TTGCACACACTAACCGCTTTCCTCGTAAATCTCACGGACACGCGTGCGTGCACCTATCCGCCTCACCCTGCACTTT 4202

**Seq. 3** 3260 TAGCTGCTTGAGAGTCTTACGTGCGTTGAGCTCCTGTAGTCCTCTATTACGCGCCGCACCCCTCTTACACGCCCGA 3335

**Seq. 4** 4203 TAGCTGCTTGAGAGTCTTACGTGCGTTGAGCTCCTGTAGTCCTCTATTACGCGCCGCACCCCTCTTACACGCCCGA 4278

**Seq. 3** 3336 G

**Seq. 4** 4280 G

**Fig. S1C. Comparison of *L.major* and *L. infantum* repeated sequences of chromosome 6.**

**Identities = 533/587 (90%)**

***L. major*** 3 TGCCGACGAAGTATCCCCCGCACGCTTGCCCCCTCCGTCTGCTGAGCCAGCGCGTGAGTGCGCACCGGGCCCC 75

***L. infantum*** 1 TGCCGACGAAGTATCGCCCACCCGCTTGCCCCCTCCTTCTGCTGAACCAGCGCGTGAGTGCGCACCGGGCCCC 73

***L. major*** 76 GGCGGCTGACATGTTTTTGGTGCCGTTCTCCTGCGCGTTCACGGAATGTCTTGCGGTTGCACGCGTTTCCGTG 148

***L. infantum*** 74 GGCGGCCGACATGTTTT-GGTGTCGTTCTCTTGCGCGTTCTTTGAATGTTTTGCGGTTGCACGCGTTTCCGTG 146

***L. major*** 149 GGCGCAACACGGCGGCGAGATCGGGTGGGCTGACCGATGGCGTCCTCGTTTCTTCGCCTCCGTGGAGGTGCG 221

***L. infantum*** 147 GACGCAACACGGCGACGAGATCAGGTGGACTGACTGATGGCGTCCTCGTTTCTTCGCCTCAGTGGAGGCGTG 218

***L. major*** 222 ACGTCTGTCCCGCCCCTCAGCGACAC--GTGTGGCCTTCTACTCTCTGTGGGCACGGCCCCCT--------- 283

***L. infantum*** 219 ACGTCTGTCCCACCTCTCAGCGATACGTGTGTGGTCTTCTACTCTCTGTGGGCACGGGCCCCCCGGTGACCG 291

***L. major*** 284 --------CCCTTCTCCCTCGTCATCTGCATGGTCCGTGCCTTCAGAGGGCGTCCGACCACGCCCCTCGTGT 348

***L. infantum*** 292 CATGGCCCCCCTTCTTCCTCGTCATCTGCATGGCCCGTGCCTTCAGAGGGCGTCCGGCCACGCCCTTCGTGT 364

***L. major*** 349 CTCCGCTTGGTGGGCAGGTAGCGGGCAGGACGAAGCGGGGGCGATGTCCCACACGCCCACGCACCGCCCAGA 421

***L. infantum*** 365 CTCCGCTCGGTGGACAGGTAGCGGGAAGGACGAAGCGGGTGCGATGGCACACACGCCCACACACCGCCCAGA 437

***L. major***  422 AGTCGGATGCCTTGCGACGAATCTGCGGACTCGCTCCGTGGCCTCGCGCTACCATGCACGCCGTCTCTGCCT 494

***L. infantum*** 438 AGTCGGATGCCTTACGACGAATGTGCTGACTCGCTCCGTGGCCTCGCGCTGCCACGCACTCCGTCTCTGCCT 510

***L. major***  495 CTCCACCGCCACTCTCTCTCTCTCTCTCTCTTCTCCCTCACACGCTGCTGAAGGAGCGAAGGTACGCGCATG 567

***L. infantum*** 511 CTCCACCGCCG----------CTCTCTCTCTTCTCCCACACACGCTGGCGAAGGAGCGAAGGTACTCGCATG 573

***L. major*** 568 CGCACTGGCCTTGT 581

***L. infantum*** 574 CATACCGGCCTTGT 587

**Fig. S1D. Recombination between direct repeated sequences in *L. major* leads to a *DHFR-TS* containing extrachromosomal circle.**

**Recomb:** sequence of the PCR fragment obtained with primers 6a and 6b (see figure 4D)

**Seq. 1**: LmjF06_01_20050601_V5.2 (Subsequence range: 343046-367401)

**Seq. 2**: LmjF06_01_20050601_V5.2 (Subsequence range: 369673-390923)

**Recomb** 1 TCGGTCGTTACGTGACGGGCATGCGCTGCTGAGCGCGGCGCGAGACATGGCAGGGCATCCGCCTTTTGAACAGTGC 76

**Seq. 2 11220** TCGGTCGTTACGTGACGGGCATGCGCTGCTGAGCGCGGCGCGAGACATGGCAGGGCATCCGCCTTTTGAACAGTGC 11295

**Recomb 77** GCCGTAGCGCAGACGGGCACCGTGATCAAGGACAGAGGTGCGTGCTTTCTTATATGTATGCGTGCGTCTGTGCATG 152

**Seq. 2** 11296 GCCGTAGCGCAGACGGGCACCGTGATCAAGGACAGAGGTGCGTGCTTTCTTATATGTATGCGTGCGTCTGTGCATG 11371

**Recomb** 153 TGTAACGAGCTCCGCGCGTGCTGCCTGCACTTGCTAGACGCTGGTTGTGTCGCCGTAGCACGTGGGCGCCCACCGC 228

**Seq. 2** 11372 TGTAACGAGCTCCGCGCGTGCTGCCTGCACTTGCTAGACGCTGGTTGTGTCGCCGTAGCACGTGGGCGCCCACCGC 11447

**Recomb** 229 AAGAGAGATGTGTGTGTGTGTGTGTGCCTGAACGCTTGATGTTGTTGTCTTGCTTCGTTTCTTTTCTTATATTTGT 304

**Seq. 2** 11448 AAGAGAGATGTGTGTGTGTGTGTGTGCCTGAACGCTTGATGTTGTTGTCTTGCTTCGTTTCTTTTCTTATATTTGT 11523

**Recomb** 305 TGTACAGGGCCTTTGCTACCCCTCCCCTTCTCGATCTCCAGAAGAGTCGATGTTCCTTCCCTTTCCATGACAGCCC 380

**Seq. 2** 11524 TGTACAGGGCCTTTGCTACCCCTCCCCTTCTCGATCTCCAGAAGAGTCGATGTTCCTTCCCTTTCCATGACAGCCC 11599

**Recomb** 381 CCCTCCCCCTTCTCCACACACACACACACACATACGTAGGTATATATATGTATATATATGTGCATGTGCATGTGTG 456

**Seq. 2** 11600 CCCTCCCCCTTCTCCACACACACACACACACATACGTAGGTATATATATGTATATATATGTGCATGTGCATGTGTG 11675

**Recomb** 457 TGTGTGTGTGTGTGTGTGGCGTGTGTGTGTGTGTGTGTGTGTGGCTGTCGGTGCTATCGCGTGAGTGCGCCCTCTC 532

**Seq. 2** 11676 TGTGTGTGTGTGTGTGTGGCGTGTGTGTGTGTGTGTGTGTGTGGCTGTCGGTGCTATCGCGTGAGTGCGCCCTCTC 11751

**Recomb** 533 CCTCCCCCCTTTTCCGTGTTCTTGCGCCTCTTTCCTTCCGAACACCTCGCTCGCTGTCACCCCACGCGGCTGGCGC 608

**Seq. 2** 11752 CCTCCCCCCTTTTCCGTGTTCTTGCGCCTCTTTCCTTCCGAACACCTCGCTCGCTGTCACCCCACGCGGCTGGCGC 11827

**Recomb** 609 CTCCTCGCTTCTCTGCTTCGCCTCCCGTTTGCTTGTTCCGTGTGTGTGTGTGTGTGTGTGTGTGTGTGTGTGTGTG 684

**Seq. 2** 11828 CTCCTCGCTTCTCTGCTTCGCCTCCCGTTTGCTTGTTCCGTGTGTGTGTGTGTGTGTGTGTGTGTGTGTGTGTGTG 11903

**Seq. 1**  GCTGCCGACGAAGTATCCCCCGCACGCTTG 9290 **Recomb** 685 TGTGTGTGCGTTCTGGTGCTCGCCTACCCCGCTTGCTTGCTTGCTTGCTGCCGACGAAGTATCCCCCGCACGCTTG 760

**Seq. 2** 11904 TGTGTGTGCGTTCTGGTGCTCGCCTACCCCGCTTGCTTGCTTGCTTGCTGCCGACGAAGTATCCCCCGCACGCTTG 11979

**Seq. 1** 9291 CCCCCTCCGTCTGCTGAGCCAGCGCGTGAGTGCGCACCGGGCCCCGGCGGCTGACATGTTTTTGGTGCCGTTCTCC 9366

**Recomb** 761 CCCCCTCCGTCTGCTGAGCCAGCGCGTGAGTGCGCACCGGGCCCCGGCGGCTGACATGTTTTTGGTGCCGTTCTCC 836

**Seq. 2** 11980 CCCCCTCCGTCTGCTGAGCCAGCGCGTGAGTGCGCACCGGGCCCCGGCGGCTGACATGTTTTTGGTGCCGTTCTCC 12055

**Seq. 1** 9367 TGCGCGTTCACGGAATGTCTTGCGGTTGCACGCGTTTCCGTGGGCGCAACACGGCGGCGAGATCGGGTGGGCTGAC 9442

**Recomb** 837 TGCGCGTTCACGGAATGTCTTGCGGTTGCACGCGTTTCCGTGGGCGCAACACGGCGGCGAGATCGGGTGGGCTGAC 912

**Seq. 2** 12056 TGCGCGTTCACGGAATGTCTTGCGGTTGCACGCGTTTCCGTGGGCGCAACACGGCGGCGAGATCGGGTGGGCTGAC 12131

**Seq. 1** 9443 CGATGGCGTCCTCGTTTCTTCGCCTCCGTGGAGGTGCGACGTCTGTCCCGCCCCTCAGCGACACGTGTGGCCTTCT 9518

**Recomb** 913 CGATGGCGTCCTCGTTTCTTCGCCTCCGTGGAGGTGCGACGTCTGTCCCGCCCCTCAGCGACACGTGTGGCCTTCT 988

**Seq. 2** 12132 CGATGGCGTCCTCGTTTCTTCGCCTCCGTGGAGGTGCGACGTCTGTCCCGCCCCTCAGCGACACGTGTGGCCTTCT 12207

**Seq. 1** 9519 ACTCTCTGTGGGCACGGCCCCCTCCCTTCTCCCTCGTCATCTGCATGGTCCGTGCCTTCAGAGGGCGTCCGACCAC 9594

**Recomb** 989 ACTCTCTGTGGGCACGGCCCCCTCCCTTCTCCCTCGTCATCTGCATGGTCCGTGCCTTCAGAGGGCGTCCGACCAC 1064

**Seq. 2** 12208 ACTCTCTGTGGGCACGGCCCCCTCCCTTCTCCCTCGTCATCTGCATGGTCCGTGCCTTCAGAGGGCGTCCGACCAC 12283

**Seq. 1** 9595 GCCCCTCGTGTCTCCGCTTGGTGGGCAGGTAGCGGGCAGGACGAAGCGGGGGCGATGTCCCACACGCCCACGCACC 9670

**Recomb** 1065 GCCCCTCGTGTCTCCGCTTGGTGGGCAGGTAGCGGGCAGGACGAAGCGGGGGCGATGTCCCACACGCCCACGCACC 1140

**Seq. 2** 12284 GCCCCTCGTGTCTCCGCTTGGTGGGCAGGTAGCGGGCAGGACGAAGCGGGGGCGATGTCCCACACGCCCACGCACC 12359

**Seq. 1** 9671 GCCCAGAAGTCGGATGCCTTGCGACGAATCTGCGGACTCGCTCCGTGGCCTCGCGCTACCATGCACGCCGTCTCTG 9746

**Recomb** 1141 GCCCAGAAGTCGGATGCCTTGCGACGAATCTGCGGACTCGCTCCGTGGCCTCGCGCTACCATGCACGCCGTCTCTG 1216

**Seq. 2** 12360 GCCCAGAAGTCGGATGCCTTGCGACGAATCTGCGGACTCGCTCCGTGGCCTCGCGCTACCATGCACGCCGTCTCTG 12435

**Seq. 1** 9747 CCTCTCCACCGCCACTCTCTCTCTCTCTCTCTCTTCTCCCTCACACGCTGCTGAAGGAGCGAAGGTACGCGCATGC 9822

**Recomb** 1217 CCTCTCCACCGCCACTCTCTCTCTCTCTCTCTCTTCTCCCTCACACGCTGCTGAAGGAGCGAAGGTACGCGCATGC 1292

**Seq. 2** 12436 CCTCTCCACCGCCACTCTCTCTCTCTCTCTCTCTTCTCCCTCACACGCTGCTGAAGGAGCGAAGGTACGCGCATGC 12511

**Seq. 1** 9823 GCACTGGCCTTGTCACACGCACACACACACACACACACACACACACACGCACACACACACACGCACGCACAGGCAC 9898

**Recomb** 1293 GCACTGGCCTTGTCACACGCACACACACACACACACACACACACACACGCACACACACACACGCACGCACAGGCAC 1368

**Seq. 2** 12512 GCACTGGCCTTGT

**Seq. 1** 9899 ACACTTTATCGGCGTCTCGGTGCGCGTGCGTCGTTGTTGCTGACCTTCGTCACGCCCGCTCTTTTCCCCACCTGCG 9974

**Recomb** 1369 ACACTTTATCGGCGTCTCGGTGCGCGTGCGTCGTTGTTGCTGACCTTCGTCACGCCCGCTCTTTTCCCCACCTGCG 1444

**Seq. 1** 9975 GCGCCCTCGCACGCCGTCGAAAAACGATGATCTCGACCCT 10014

**Recomb** 1445 GCGCCCTCGCACGCCGTCGAAAAACGATGATCTCGACCCT 1484

**Figure S1:** The extrachromosomal DHFR-TS circular amplicon is generated by the homologous recombination of two direct repeated sequences of 575 bp in *L. major* (**A**) and 837 bp in *L. infantum* (**B**). These repeated sequences are highly similar between the two species (**C**). The sequence of the PCR generated amplicon from mutant *L. major* MTX60.4 confirmed the scenario of homologous recombination in *L. major* (**D**) and *L. infantum* (not shown). The repeated sequence in D is in gray. The boxed sequences correspond to the 3’end of *LmjF06.0880* and underlined sequence to the ATG of *LmjF06.0830* (see also Fig. 4). The sequence can be found under accession number EU346088

**Fig S2A. Chromosome 23 inverted repeated sequences in *L. infantum*.**

**Seq. 5**: LinJ23_20070420_V3 (Subsequence range: 118249-130000)

**Identities = 575/581 (98%), Gaps = 3/581 (0%)**

**Seq. 5 (+)** 1247 ATGCGTCCATTTCTCTGTCGTAGAACACCATGAGTTCAGCTTCCCTTCCACTCTGCTCTGCGGCCCTGCC 1316

**Seq. 5 (-)** 8674 ATGCGCCCATTTCTCTGTCG---AACACCATGAGTTCAGCTTCCCTTCCACTCTGCTCTGCAGCCCTGCC 8608

**Seq. 5 (+)** 1317 GCAGGCCCCACACCGCGCGGTGCGAAGCAGCCGTCGACACAAGCGTTGCAGCAGTGCGCCGGCCCAGCCA 1386

**Seq. 5 (-)** 8607 GCAGGCCCCACACCGCGCGGTGCGAAGCAGCCGTCGACACAAGCGTTGCAGCAGTGCGCCGGCCCAGCCA 8538

**Seq. 5 (+)** 1387 ACTGAGAGCATGAGCTCCTCATCAAACTCTACCCACCAAAACACCGCGTTGCAGCCGCTCCTTCCATCAT 1456

**Seq. 5 (-)** 8537 ACTGAGAGCATGAGCTCCTCATCAAACTCTACCCACCAAAACACCGCGTTGCAGCCGCTCCTTCCATCAT 8468

**Seq. 5 (+)** 1457 GCCGGTCTCCACCCCTGGTACATCCCCCTCGGGGTGACACGCAGGCTCCCCTCCACACCAGCAAGCCGTG 1526

**Seq. 5 (-)** 8467 GCCGGTCTCCACCCCTGGTACATCCCCCTCGGGGTGACACGCAGGCTCCCCTCCACACCAGCAAGCCGTG 8398

**Seq. 5 (+)** 1527 TGAGGGCCGGGAGGGGGATACGCTGGAGTCACGCGGACACTCTGCCTATCACATGGATGGCACAAGCGTG 1596

**Seq. 5 (-)** 8397 TGAGGGCCGGGAGGGGGATACGCTGGAGTCACGCGGACACTCTGCCTATTACATGGATGGCACAAGCGTG 8328

**Seq. 5 (+)** 1597 CTCTCTGCCGCGTGTCGCTCTCCGACGCACCCCCATCCGGGACCTCACCGCCGACATCAGCAGCGATGGC 1666

**Seq. 5 (-)** 8327 CTCTCTGCCGCGTGTCGCTCTCCGACGCACCCCCATCCGGGACCTCACCGCCGACATCAGCAGCGATGGC 8258

**Seq. 5 (+)** 1667 TCCCACGGACCTCGCCATGTCGTAGGCACGCGACCCTCTGACCAACAGAGATGTGGTTCAGCATGAGCCG 1736

**Seq. 5 (-)** 8257 TCCCACGGACCTCGCCATGTCGTAGGCACGCGACCCTCTGACCAACAGAGATGTGGTTCAGCATGAGCCG 8188

**Seq. 5 (+)** 1737 GGAATAGGATGGCCGCCTGGAATCTCCCCACCGAGAGCAGGCGCACTGGTCCTTGAAGCCATGCACTGAG 1806

**Seq. 5 (-)** 8187 GGAATAGGATGGCCGCCTGGAATCTCCCCACCGAGAGCAGGCGCACTGGTCCTTGAAGCCATGCACTGAG 8118

**Seq. 5 (+)** 1807 AGGTGTCCTGCGTCATCAAGG 1827

**Seq. 5 (-)** 8117 AGGTGTCCTGCGTCATCAAGG 8097

**Fig. S2B. Alignment of the linear amplicon related PCR fragment with chromosome 23.**

**Recomb :** Sequence of the PCR fragment 23ab (see Fig. 5D)

**Seq. 5 (+)**: **+** strand LinJ23_20070420_V3 (Subsequence range: 118249-130000)

**Seq. 5 (-):** - strand LinJ23_20070420_V3 (Subsequence range: 118249-130000)

**Seq. 5 (+)** 833 AGCGGTGAAGGGGTGGGTGGGTGGAGAGCATGCGAGTGCGTCGACGCGATGCATGGAGCAGGCTGATGTG 902

**Recomb** 1 AGCGGTGAAGGGGTGGGTGGGTGGAGAGCATGCGAGTGCGTCGACGCGATGCATGGAGCAGGCTGATGTG 70

**Seq. 5 (+)** 903 GATGAGTCGCTAACTCCGCAATCCCGCCCTCGCTGCGTTTTCTCCTCAGTCGATGCGCCTGTACGTGTGT 972

**Recomb** 71 GATGAGTCGCTAACTCCGCAATCCCGCCCTCGCTGCGTTTTCTCCTCAGTCGATGCGCCTGTACGTGTGT 140

**Seq. 5 (+)** 973 CCATGGAAGGAGACGCGTTGTTGCGGGAGTGGGGTGGAAGGGAGGGAGATCTGAAATGGTACGCGCAAGC 1042

**Recomb** 141 CCATGGAAGGAGACGCGTTGTTGCGGGAGTGGGGTGGAAGGGAGGGAGATCTGAAATGGTACGCGCAAGC 210

**Seq. 5 (+)** 1043 ATACTCGCATGCTTGTTTCCTCCCACTGCCAACGTTATAGCAACTGCCGTCACCCCGACGCCGCCCACTC 1112

**Recomb** 211 ATACTCGCATGCTTGTTTCCTCCCACTGCCAACGTTATAGCAACTGCCGTCACCCCGACGCCGCCCACTC 280

**Seq. 5 (+)** 1113 CCGCCTCGATGCCCTCTCCTGACCCCCCCACCCCCACCCTTTGCCTGTACACGTGCCTGGGTCAGTGAAT 1182

**Recomb** 281 CCGCCTCGATGCCCTCTCCTGACCCCCCCACCCCCACCCTTTGCCTGTACACGTGCCTGGGTCAGTGAAT 350

**Seq. 5 (+)** 1183 AGAGGGCTGCCAACATTGTTTCGCTAAGCTTAGCATCGAGGACATCAGCTTTTCAAATTACTTCATGCGC 1252

**Recomb** 351 AGAGGGCTGCCAACATTGTTTCGCTAAGCTTAGCATCGAGGACATCAGCTTTTCAAATTACTTCATGCGC 420

**Seq. 5 (-)**  ATGCGT 8669

**Seq. 5 (+)** 1253 CCATTTCTCTGTCG---AACACCATGAGTTCAGCTTCCCTTCCACTCTGCTCTGCAGCCCTGCCGCAGGC 1319

**Recomb** 421 CCATTTCTCTGTCG---AACACCATGAGTTCAGCTTCCCTTCCACTCTGCTCTGCAGCCCTGCCGCAGGC 487

**Seq. 5 (-)** 8668 CCATTTCTCTGTCGTAGAACACCATGAGTTCAGCTTCCCTTCCACTCTGCTCTGCGGCCCTGCCGCAGGC 8599

**Seq. 5 (+)** 1320 CCCACACCGCGCGGTGCGAAGCAGCCGTCGACACAAGCGTTGCAGCAGTGCGCCGGCCCAGCCAACTGAG 1389

**Recomb** 488 CCCACACCGCGCGGTGCGAAGCAGCCGTCGACACAAGCGTTGCAGCAGTGCGCCGGCCCAGCCAACTGAG 557

**Seq. 5 (-)** 8598 CCCACACCGCGCGGTGCGAAGCAGCCGTCGACACAAGCGTTGCAGCAGTGCGCCGGCCCAGCCAACTGAG 8529

**Seq. 5 (+)** 1390 AGCATGAGCTCCTCATCAAACTCTACCCACCAAAACACCGCGTTGCAGCCGCTCCTTCCATCATGCCGGT 1459

**Recomb** 558 AGCATGAGCTCCTCATCAAACTCTACCCACCAAAACACCGCGTTGCAGCCGCTCCTTCCATCATGCCGGT 627

**Seq. 5 (-)** 8528 AGCATGAGCTCCTCATCAAACTCTACCCACCAAAACACCGCGTTGCAGCCGCTCCTTCCATCATGCCGGT 8459

**Seq. 5 (+)** 1460 CTCCACCCCTGGTACATCCCCCTCGGGGTGACACGCAGGCTCCCCTCCACACCAGCAAGCCGTGTGAGGG 1529

**Recomb** 628 CTCCACCCCTGGTACATCCCCCTCGGGGTGACACGCAGGCTCCCCTCCACACCAGCAAGCCGTGTGAGGG 697

**Seq. 5 (-)** 8458 CTCCACCCCTGGTACATCCCCCTCGGGGTGACACGCAGGCTCCCCTCCACACCAGCAAGCCGTGTGAGGG 8389

**Seq. 5 (+)** 1530 CCGGGAGGGGGATACGCTGGAGTCACGCGGACACTCTGCCTATTACATGGATGGCACAAGCGTGCTCTCT 1599

**Recomb** 698 CCGGGAGGGGGATACGCTGGAGTCACGCGGACACTCTGCCTATCACATGGATGGCACAAGCGTGCTCTCT 767

**Seq. 5 (-)** 8388 CCGGGAGGGGGATACGCTGGAGTCACGCGGACACTCTGCCTATTACATGGATGGCACAAGCGTGCTCTCT 8319

**Seq. 5 (+)** 1600 GCCGCGTGTCGCTCTCCGACGCACCCCCATCCGGGACCTCACCGCCGACATCAGCAGCGATGGCTCCCAC 1669

**Recomb** 768 GCCGCGTGTCGCTCTCCGACGCACCCCCATCCGGGACCTCACCGCCGACATCAGCAGCGATGGCTCCCAC 837

**Seq. 5 (-)** 8318 GCCGCGTGTCGCTCTCCGACGCACCCCCATCCGGGACCTCACCGCCGACATCAGCAGCGATGGCTCCCAC 8249

**Seq. 5 (+)** 1670 GGACCTCGCCATGTCGTAGGCACGCGACCCTCTGACCAACAGAGATGTGGTTCAGCATGAGCCGGGAATA 1739

**Recomb** 838 GGACCTCGCCATGTCGTAGGCACGCGACCCTCTGACCAACAGAGATGTGGTTCAGCATGAGCCGGGAATA 907

**Seq. 5 (-)** 8248 GGACCTCGCCATGTCGTAGGCACGCGACCCTCTGACCAACAGAGATGTGGTTCAGCATGAGCCGGGAATA 8179

**Seq. 5 (+)** 1740 GGATGGCCGCCTGGAATCTCCCCACCGAGAGCAGGCGCACTGGTCCTTGAAGCCATGCACTGAGAGGTGT 1809

**Recomb** 908 GGATGGCCGCCTGGAATCTCCCCACCGAGAGCAGGCGCACTGGTCCTTGAAGCCATGCACTGAGAGGTGT 977

**Seq. 5 (-)** 8178 GGATGGCCGCCTGGAATCTCCCCACCGAGAGCAGGCGCACTGGTCCTTGAAGCCATGCACTGAGAGGTGT 8109

**Seq. 5 (+)** 1810 CCTGCGTCATCAAGG

**Recomb** 978 CCTGCGTCATCAAGGGATGAAATGAAGCTGGAAAGCGGAGGAGATGAGCAGACAGAGACACACGACGGCA 1047

**Seq. 5 (-)** 8108 CCTGCGTCATCAAGGGATGAAATGAAGCTGGAAAGCGGAGGAGATGAGCAGACAGAGACACACGACGGCA 8039

**Recomb** 1048 CGCACTCACGCAAGCCAGCACACGGACTAGATCAAAAGCAGAACGATGGCGCCGAGGAAAGACAGCGATG 1117

**Seq. 5 (-)** 8038 CGCACTCACGCAAGCCAGCACACGGACTAGATCAAAAGCAGAACGATGGCGCCGAGGAAAGACAGCGATG 7969

**Recomb** 1118 ATTTCGCAAAGTCCGTTGTCGGCATGCGAAATAAAAAAGACGGTGCCAAATCACATACGATAAATCAGAA 1187

**Seq. 5 (-)** 7968 ATTTCGCAAAGTCCGTTGTCGGCATGCGAAATAAAAAAGACGGTGCCAAATCACATACGATAAATCAGAA 7899

**Recomb** 1188 GTGTAGACGGACTGCAATCTTATACACGTAAACCTACATATATATACAGAGGTGTACTTTTATACTTGCA 1257

**Seq. 5 (-)** 7898 GTGTAGACGGACTGCAATCTTATACACGTAAACCTACATATATATACAGAGGTGTACTTTTATACTTGCA 7829

**Recomb** 1258 GACGTGTCCGCGTCTCTGCCCGTGTCTAGCAGCTGACGGGAAGGGGTCCCACAGAGAGAGAGAAGGGCAT 1327

**Seq. 5 (-)** 7828 GACGTGTCCGCGTCTCTGCCCGTGTCTAGCAGCTGACGGGAAGGGGTCCCACAGAGAGAGAGAAGGGCAT 7759

**Recomb** 1328 ACAGCACAGGATAAAGAGAAAGAGAGGGTGCCCAGAGATGGACAGCAGGCCCCTCAAGGTGTGCGGAGCT 1397

**Seq. 5 (-)** 7758 ACAGCACAGGATAAAGAGAAAGAGAGGGTGCCCAGAGATGGACAGCAGGCCCCTCAAGGTGTGCGGAGCT 7689

**Recomb** 1398 GAGGATGTAAAAGAAACGACAAAACAGCGGAACATGAACGATGCAAGTAACAGAGGAGCAGCGCACAGAG 1467

**Seq. 5 (-)** 7688 GAGGATGTAAAAGAAACGACAAAACAGCGGAACATGAACGATGCAAGTAACAGAGAGCAGCGCACAGAGG 7619

**Recomb** 1468 AGCAAGCATGCGCAAGCGTGCGCTGAAGCTCACAGATGCGGTGGGAGAGAGAGACAGGGGAAGGGTGTGG 1537

**Seq. 5 (-)** 7618 AGCAAGCATGCGCAAGCGTGCGCTGAAGCTCACAGATGCGGTGGGAGAGAGAGACAGGGGAAGGGTGTGG 7549

**Recomb** 1538 ATGGGGCGGGAAGAAGCCATTGTCGGCAGCGTGGACGCGGCGTTGTGCGAGTGCGTGTGTTTCTGTGACG 1607

**Seq. 5 (-)** 7548 ATGGGGCGGGAAGAAGCCATTGTCGGCAGCGTGGACGCGGCGTTGTGCGAGTGCGTGTGTTTCTGTGACG 7479

**Recomb** 1608 GTGTGTGGTTCATAGGAGATAGGTGGGCACGGGTGCGTTGTTGCCTGCTCGGCCCCGTA 1550

**Seq. 5 (-)** 7478 GTGTGTGGTTCATAGGAGATAGGTGGGCACGGGTGCGTTGTTGCCTGCTCGGCCCCGTA 7420

**Figure S2:** The extrachromosomal 230 kb linear amplicon is generated by the homologous recombination of two inverted repeated sequences (**A**) on chromosome 23. The model proposed for inverted duplication (see Fig. 5C) is supported by the analysis of the recombined sequence isolated by PCR using the primer pair 23a/23b (**B**). The repeated sequences are shaded in gray. The sequence of the new junction can be found under accession number EU346089.

**Figure S3. Sequence of the *L. major* chimera gene *LmjF10.0380/0390***. The amplified fragment derived from *L. major* MTX60.4 obtained using primers F and R (indicated as lower case letters) was sequenced. The coding sequence (captial letters, starting from ATG) of *LmjF10.0380* is shaded in black, the conserved sequence (75bp) used for rearrangement of *LmjF10.0380* and *10.0390* is underlined, and the sequence corresponding to *LmjF10.0390* (until the stop codon) is shaded in gray. This sequence has the accession number EU346090.

ggcatcagtgaagtggaaccATGTCCCACAAGGAGGCCGCTCCCAAGCGCGAGAAGGATGCCGCCTCAGACGCCGCGGGAAATGATGACAAGTACATCCACCCCGAGGCAGCATCCCTGTTCGCCAGGTGCCCGTGGGCTCGCCGCGTCCCCGTGTTTGGTGACGCTGTCGAGGGCTACGGGCTCAAGGTCATCGTCACTCTTGGTACGAGCTACCTGCTCTGCAAGGGTATCGCGGATCAGATTCTATATGATCAGACGTACGCCATGATGATTGATCGCTACGGCATCGACGTGGCCCGCTACCAGCGCCTGTCTCCGATTTTGTTCATGGGGTGGTCCATCAAGGCCTTCACCGCGATGCTCTGCGACGGCTTCGCCTTCCTCGGCTACACGAAGCGCTGGTACATGTTCATCTCCTGCGTCGGCGGTGGTGCGTTCGCGCTGATCTACGGCCTGCTGCCCGCGAAGGAGGCGTCGGCTGATGTGGCGTGTGCCTTCATCTTCCTGTCGTGCTGGGGCAAGGCCAACGTGGATATCCTGTCCCAGGGCCATTACAGTCGACTGATGCGCGAGAACCCGAAGCCTGGCCCGGCGCTGGTGAGCTGGATCTGGCTCTGGATCATGACCGGCTCGCTCATCGCGACTGTGATGAACGGCCCGCTCGCGGATGCCGGGAAGCCGCAGATCAGCATCTTCGTGTCTGCCGCGCTGCAGGCCATCACCTGCGTCTTCTACCTGTTCAACTGGTACGGGGAGAAGAAGAACCGCGTGCTGCGCTCCGAGGACGCGCTGTTTATTCTGGAGGAGACCCGCAAGGAGCGTGAGCGCCTGGGTCTCGAGGGGGTGTACGACGGCACGGCGGGTGCGCAGCATGGTGGTGCGGCGAAGGGGAAGAAGAGCCCGCAGCACTCGCACTCGGATGAGGACGTGGAGGGTGCCGTACGGGACGCCCTCAACGATGGTCAGCGCGACAACGGTGAACTTGTGCAGGACGTCTACGACGACGCGTATGACGACGGCGAGGGGGTGGCCGAGGGCGATGTGTACTACGGCAAGCCGCCGGTGCCGTGCCTGTTCGGGCTGTTCGAGGCAAACACGGAGGTGATTTCGAAGAACTGGAAGATCTTCGTGTACAGCGTCGTCATGACCTGTGCTGTGATCACGATGCTGTGTGCCAACATGCTGGCCGACACGCTGGGCCTCCTGGTTGCGTGCGTCGTTGTGTCGACCATCTGCTGTACCACGTCCTTCTGGGCCCTGCCGCTGGTGATTGCGAAGGCCAACGTGTTTGCATACCTGGATAAGGCTGTTTCCATCCGTGTGGGCGGTCCCCTAAATGCGTTCTACTTGAACACCTACCAGTGCCCTGGCAACCTGCCGAACTTCACCTACACCTTCTACAACACGGTGGCGGGCGTGATTAGCAGTGCTGTTGGTGTGATTACCGTGACGCTGTTCAACTTCCTGTTCGCGAAGCATGGCTACTGCCTCACCTTCATTGTGACGACAATCATGCAGGTTATGGGTGGTGTGTTCGACATCATCATTGTGAAGCGGTGGAACCTGTACATTGGCATCCCTGACCACGCCATGTACATCTGGGGTGATGCTGTTGTGGGTGAGCTCGTGTACATGCTTGGCTTTATGCCGCAGATCGTGCTGCTGTCTCGCCTGTGCCCTCGTGGCTCGGAGAGTGTCGTGTATGCGCTGATGGCGGGCTTCGCGAGTCTTGGCCAGACCACCGCGTCGTCCCTCGGTGCGATCATCATGGAGTACGGCCTGCCTGTGTTCAAGACCCAGGATGACGGGTCTCGCTGCAACTACGACAACCTGCCGCTGCTGCTGTTCCTGTGCAGCATGTGCACGCCGCTGCTGGTGATTCCGCTGAGCATGATACTGCTTCCGAAGGCGCGCATCTGCGACGATATCGACGTTGACGGCAAGGTGGTGCGCCAGGCCGTGGATAAGCAGGTCGCAGCTGCTCCGCTGTCGAGCTCCGACTCGGACGCGGTGATGGCTGCCGAGCCGCTTCATGGGAACAAGGCGGATGAGCGCGAGGCAGCGCGCGGGGAGGCGGTGTAGggcaacgcgtgaagcagaagcc
