# Supplementary figures and images for: Modulation of gene expression in drug resistant Leishmania is associated with gene amplification, gene deletion and chromosome aneuploidy
Source: Genome Biol. 2008 Jul 18;9(7):R115. doi: 10.1186/gb-2008-9-7-r115 (PMC2530873; doi:10.1186/gb-2008-9-7-r115)

## Slide 1
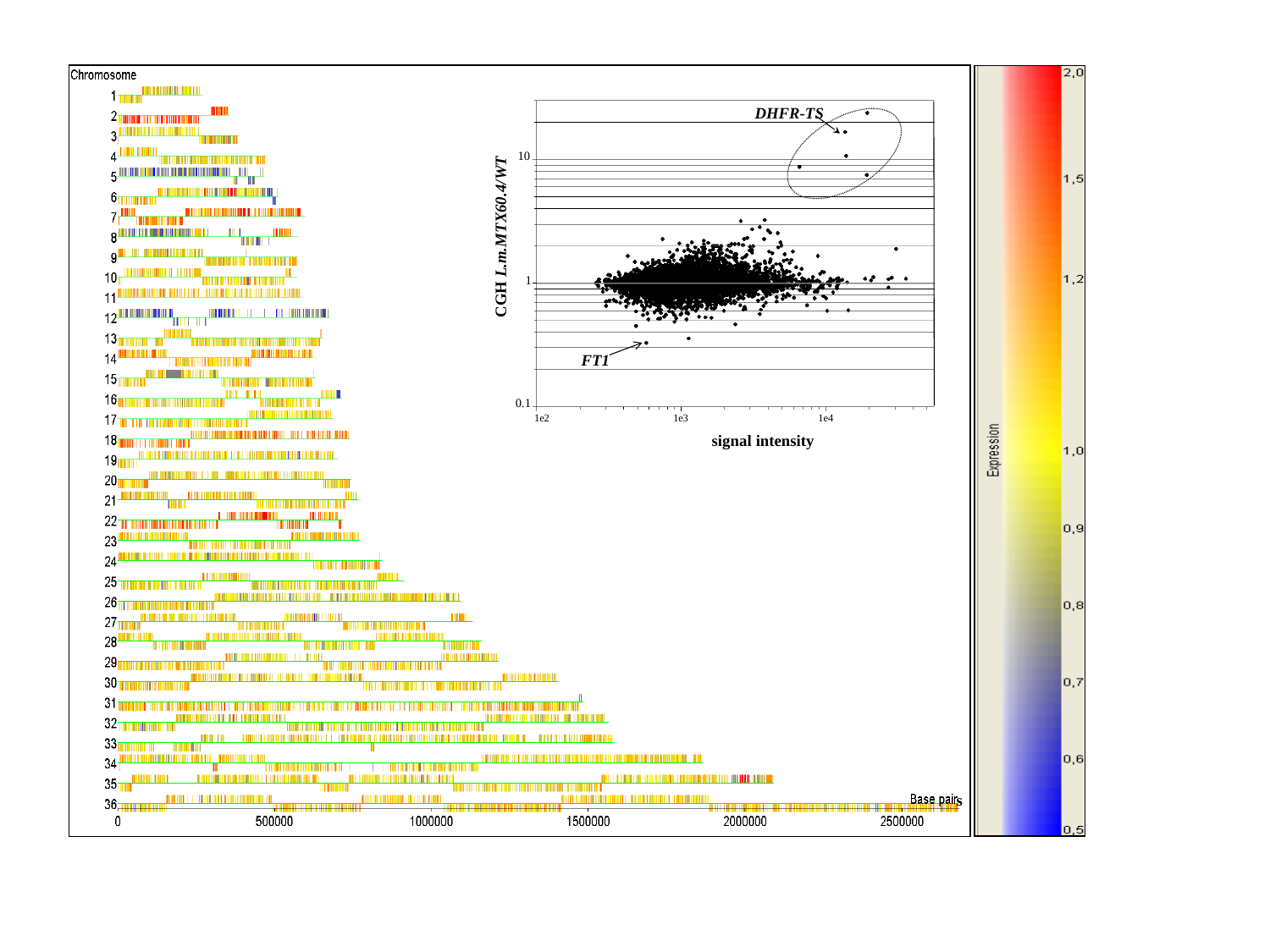

s
DHFR-TS
10
CGH L.m.MTX60.4/WT
1
FT1
0.1
1e2
1e3
1e4
signal intensity

Supplement: Additional data file 3 — Results of the comparative genomic hybridization analyses of L. major MTX60.4 versus the respective wild-type cells. [file gb-2008-9-7-r115-S3.ppt]
